# Oncogenic RAS drives the CRAF‐dependent extracellular vesicle uptake mechanism coupled with metastasis

**DOI:** 10.1002/jev2.12091

**Published:** 2021-06-10

**Authors:** Dongsic Choi, Laura Montermini, Brian Meehan, Anthoula Lazaris, Peter Metrakos, Janusz Rak

**Affiliations:** ^1^ Department of Biochemistry College of Medicine Soonchunhyang University Cheonan Chungcheongnam Republic of Korea; ^2^ Research Institute of the McGill University Health Centre Glen Site McGill University Montreal Quebec Canada; ^3^ Cancer Research Program, Research Institute of the McGill University Health Centre Glen Site McGill University Montreal Quebec Canada; ^4^ Department of Surgery Research Institute of the McGill University Health Centre Glen Site McGill University Montreal Quebec Canada

**Keywords:** extracellular vesicles, macropinocytosis, metastasis, NHE, RAF, RAS, uptake

## Abstract

Oncogenic RAS impacts communication between cancer cells and their microenvironment, but it is unclear how this process influences cellular interactions with extracellular vesicles (EVs). This is important as intercellular EV trafficking plays a key role in cancer invasion and metastasis. Here we report that overexpression of mutant RAS drives the EV internalization switch from endocytosis (in non‐transformed cells) to macropinocytosis (in cancer cells) resulting in enhanced EV uptake. This process depends on the surface proteoglycan, fibronectin and EV engulfment mechanism regulated by CRAF. Both mutant RAS and activated CRAF expression is associated with formation of membrane ruffles to which they colocalize along with actin, sodium‐hydrogen exchangers (NHEs) and phosphorylated myosin phosphatase (pMYPT). RAS‐transformed cells internalize EVs in the vicinity of ruffled structures followed by apparent trafficking to lysosome and degradation. NHE inhibitor (EIPA) suppresses RAS‐driven EV uptake, along with adhesion‐independent clonal growth and experimental metastasis in mice. Thus, EV uptake may represent a targetable step in progression of RAS‐driven cancers.

## 1 INTRODUCTION

Activation of oncogenic pathways in cancer deregulates multiple mechanisms of intercellular communication (Choi et al., 2017). An emerging element of this interactive network is the exchange of extracellular vesicles (EVs), spherical, membrane‐bound cellular fragments carrying a cargo of lipids, proteins and nucleic acids (RNA, DNA) (van Niel et al., 2018). Cells produce EVs of varying sizes (30 nm to over 2 μm), molecular compositions and biological activities, a diversity attributed to the spectrum of underlying biogenetic processes including outward budding of the cellular plasma membrane (microvesicles), and formation of intra‐endosomal vesicles (exosomes), each coupled with still poorly understood cargo loading mechanisms (Leidal, 2020; Mathieu et al., 2019; van Niel et al., 2018). The resulting mosaics of EV populations interact with a range of potential cellular targets impacting their biological properties (Choi et al., 2019).

EVs interact with target cells through several mechanisms, including ligand‐dependent surface‐to‐surface contacts, membrane fusion, or EV engulfment processes, such as endocytosis, macropinocytsis or phagocytosis (French et al., 2017; Mulcahy et al., 2014). The nature, extent and specificity of these interactions are influenced by the properties of EVs and their cellular recipients (Murphy et al., 2019). Similarly, complex and variable are determinants of EV fate post internalisation, which may include confinement in the endosome, re‐emission, lysosomal degradation, or cargo release into the endoplasmic reticulum (ER). The latter may be required for biological activity of some of the EV associated macromolecules (Murphy et al., 2019).

Malignant transformation profoundly alters all aspects of EV‐mediated communication (Choi et al., 2017) including EV biogenesis (Al‐Nedawi et al., 2008), cargo assembly (McKenzie et al., 2016), content (Figure S1 and Table S1) (Chennakrishnaiah et al., 2020) and uptake (Kamerkar et al., 2017; Lee et al., 2016; Nakase et al., 2015). This is important as cancer‐derived EVs may carry oncogenes and potent biological mediators capable of altering the phenotypes of recipient cells (Al‐Nedawi et al., 2008), their growth (Broekman et al., 2018; Lee et al., 2016), onset of angiogenesis (Sato et al., 2019), cancer‐associated thrombosis (Geddings & Mackman, 2013), invasion (Sung et al., 2015), metastasis (Hoshino et al., 2015) and other crucial processes (Verweij et al., 2019).

While alterations in cancer cell secretome (including EV release) attracted considerable attention, less is known about whether and how oncogenic pathways distort cellular uptake and utilization of extracellular material (Commisso et al., 2013), particulate debris (Kim et al., 2018) and EVs (Lee et al., 2016; Nakase et al., 2015), all of which may influence malignant behaviour of cancer cells. The relevance of this question is highlighted by the observation that oncogenic epidermal growth factor receptor VIII (EGFRvIII) and other transforming mutations alter the EV uptake by glioma cells (Choi et al., 2018) and astrocytes (Lee et al., 2016), while in epithelial cancer cells oncogenic activation of EGFR, HRAS, KRAS, or SRC similarly upregulate EV internalization and delivery of therein encapsulated regulatory molecules and therapeutics (Kamerkar et al., 2017; Lee et al., 2016; Nakase et al., 2015).

In this regard, activation of the RAS pathway emerges as a paradigm and signalling node in the EV uptake machinery in cancer cells (Lee et al., 2016; Nakase et al., 2015), albeit with poorly understood role, mechanisms and essential effectors. This is an important gap given the fact that oncogenic RAS mutations are found over 30% of all human cancers (Prior et al., 2012), and RAS activation is even more common along with highly prevalent and consequential involvement of RAS downstream effector pathways (RAF/MAPK, PI3K/AKT) across the spectrum of human malignancies (Li et al., 2018). How RAS signalling impacts the EV uptake by cancer cells remains unclear.

Here we report that the elevated EV uptake by RAS‐transformed cells, unlike in their isogenic non‐transformed counterparts, is dependent on CRAF activity, recapitulated by the expression of activated CRAF and obliterated by pharmacological blockade of sodium‐hydrogen exchangers (NHEs) required for macropinocytosis. RAS, CRAF, its targets (pMYPT) and NHEs are co‐expressed in membrane ruffles triggered in RAS‐transformed cells, which are proximal to the apparent sites of the cellular EV entry. Blockade of this mechanism in RAS‐transformed cells by treatment with macropinocytosis inhibitor impairs their colony formation and metastasis. Thus, EV uptake may play a role in the crucial ability of RAS‐transformed cells to form clonal outgrowths.

## 2 MATERIAL AND METHODS

### 2.1 Cell lines

RAS3 cells derived from normal rat intestinal epithelial cell line (IEC18) carry the human V12 c‐H‐ras (V12HRAS) (Buick et al., 1987; Rak et al., 1995) oncogene (Lee et al., 2014, 2016). IEC18 derived Clone 25 cells carry the same mutant HRAS sequence expressed under the control of a dexamethasone inducible promoter (Rak et al., 1995). All IEC18‐derived cells were grown in culture media containing alpha MEM medium (AMEM) (Wisent, Canada) supplemented with 5% heat‐inactivated foetal bovine serum (FBS) (Wisent), 20 mM D‐glucose (Gibco, Grand Island, NY, USA), 4 mM L‐glutamine (Gibco), 10 μg/ml insulin (Wisent), and 1% penicillin‐streptomycin (Gibco) at 37°C in 5% CO_2_. MCF10A and MCF10AT were grown in Dulbecco's modified Eagle's medium‐F12 (DMEM/F12) (Sigma, St. Louis, MO, USA) supplemented with 5% horse serum (Wisent), 0.5 μg/ml hydrocortisone (Sigma), 100 ng/ml cholera toxin (Sigma), 10 μg/ml insulin, 20 ng/ml EGF (Gibco), and 1% penicillin/streptomycin at 37°C in 5% CO_2_. A431, cells were obtained from the American Type Culture Collection (ATCC, Manassas, VA), while DKS8, DLD1, and DKO1 cell lines were a generous gift from Dr. Senji Shirasawa (Kyushu University, Fukuoka, Japan) (Rak et al., 1995; Shirasawa et al., 1993). These cells were grown in Dulbecco's modified essential medium (DMEM) (Wisent) supplemented with 10% heat‐inactivated FBS and 1% penicillin‐streptomycin at 37°C in 5% CO_2_. All cell lines routinely tested negative for *Mycoplasma* contamination.

### 2.2 Isolation of EVs

EVs were purified by ultracentrifugation as described, and routinely validated for numbers and size distribution (nanotracking analysis, NTA) and periodically visualized by electron microscopy (Chennakrishnaiah et al., 2018; Lee et al., 2014, 2016). The conditioned medium was collected from cells grown for 72‐h in culture media containing EV‐depleted FBS (acquired from the supernatant after the ultracentrifugation of FBS at 150,000*g* for 18‐h at 4°C with 0.2 μm filtration). Cell debris was pre‐cleared at 400*g* for 10 min and then supernatant was passed through the 0.2 μm pore‐size syringe filter (VWR, Seattle, WA, USA). The resulting filtrate was ultracentrifuged at 110,000*g* for 1‐h and the resulting pellet was resuspended in PBS. For the labelling of EVs with PKH26 lipophilic membrane dye (Sigma), cells were treated according to the manufacturer's instructions. PKH26‐labeled cells were grown for 72‐h in culture media containing EV‐depleted FBS. To strip the surface‐associated proteins from EVs, 500 μl of isolated PKH26‐labeled EV were washed with 7.5 ml of 1 M KCl for 30 min at 4°C and further isolated by ultracentrifugation at 110,000*g* for 1‐h. The protein concentration of EVs was determined by BCA assay (Pierce Biotechnology, Rockford, IL, USA). The concentration and size of EVs were measured by NTA using NanoSight NS500 instrument containing 532 nm laser (NanoSight Ltd., UK). Three recordings of 30‐s at 37°C were captured with camera level 15 and processed with detection threshold of 5 and blur size auto by NTA software (version 3.0)

### 2.3 Western blotting

For the whole cell lysate (WCL), cells were washed twice in ice‐cold PBS and collected in lysis buffer with 50 mM Tris (pH 7.5), 1% NP‐ 40, 0.25% Na‐Deoxycholate, 100 mM NaCl, 1 mM EDTA, 1 mM Na_3_VO_4_, 1 mM NaF, and protease inhibitor cocktail (Roche, Mannheim, Germany). The protein concentration of WCL was determined by BCA assay. Proteins were resolved by SDS‐PAGE and then transferred to a polyvinylidene difluoride membrane. The membrane was blocked, incubated with primary antibody followed by the secondary antibody conjugated with horseradish peroxidase, and subjected to enhanced chemiluminescence for detection. All images were acquired by a ChemiDoc MP imager (Bio‐Rad, Hercules, CA, USA). Rabbit anti‐RAS (catalog no. 3339), rabbit anti‐ERK1/2 (catalog no. 9102), rabbit anti‐Phospho‐ERK1/2 (Thr202/Tyr204) (catalog no. 9101), and goat anti‐rabbit IgG (catalog no. 7074S) reagents were purchased from Cell Signaling Technology (Beverly, MA, USA). Goat anti‐mouse IgG (catalog no. #1706516) is from Bio‐Rad. Mouse anti‐actin (catalog no. A5441) is from Sigma. Mouse anti‐ALIX (catalog no. 611621) is from BD Biosciences (San Diego, CA, USA). Rabbit anti‐CD9 (catalog no. ab92726), rabbit anti‐GAPDH (catalog no. ab9485), and rabbit anti‐fibronectin (catalog no. ab2413) are from Abcam (Cambridge, MA, USA). Mouse anti‐CRAF antibody (catalog no. MAB4540) is from R&D Systems (Abingdon, UK). Rabbit anti‐MEK1/2 (catalog no. PA5‐31917) and rabbit anti‐Phospho‐ MEK1/2 (Ser222) (catalog no. 44–452) are from Thermo Scientific (Carlsbad, CA, USA).

### 2.4 Inhibitor treatment for EV uptake

Cells were plated in 12‐well plate at a concentration of 50,000 cells/ml overnight. Growth media was then replaced with 1 ml of culture media containing the indcated inhibitor and incubated for 1‐h. The following inhibitors were used: 50 μM EIPA (Sigma, A3085), 10 μM cytochalasin D (Sigma, C8273), 1 μM GW5074 (Sigma, G6416), rocaglamide (Sigma, SML0656), 10 μM Trametinib (Selleckchem, Houston, TX, USA, S2673), 10 μM Selumetinib (Selleckchem, S1008), 20 μM PD98059 (Calbiochem, San Diego, CA, USA, 513000), 10 μM SCH772984 (Selleckchem, S7101), 50 μM LY264002 (Selleckchem, S1105), 500 nM Wortmannin (Selleckchem, S2758), 50 μM RBC8 (Sigma, SML1295), 10 μg/ml heparin sodium (Selleckchem, S2724), 200 nM or 1 μM RGD peptide (Selleckchem, S8008), and 200 nM or 1 μM HYD‐1 peptide (KIKMVISWKG) (Sigma). PKH26‐labeled EVs were applied to cells at 5 × 10^9^ particles/ml for 18‐h and then the transferred PKH26 fluorescent signal was quantified by flow cytometry BD FACSCalibur (BD Biosciences).

### 2.5 Flow cytometry for 70 kDa dextran and transferrin uptake

Cells were plated in 12‐well plate at a concentration of 50,000 cells/ml overnight. For IEC18 and RAS3 cells, 12.5 μg/ml of 70 kDa dextran‐Oregon Green 488 (Thermo Scientific) or 12.5 μg/ml of transferrin‐Alexa Fluor 488 (Thermo Scientific) were incubated for 18‐h. For DKO1 and DKS8 cells, 1 ml of culture media with or without 50 μM of EIPA was pre‐incubated for 1‐h and then 20 μg/ml of 70 kDa dextran‐Oregon Green 488 or 20 μg/ml of transferrin‐Alexa Fluor 488 were incubated for 18‐h with or without 50 μM of EIPA. The transferred fluorescent signal was quantified by flow cytometry BD FACSCalibur for IEC18 and RAS3 cells and BD LSRFortessa for DKO1 and DKS8 cells.

### 2.6 Confocal microscopy

Cells were plated on μ‐Slide 8‐Well ibiTreat chambered coverslips (ibidi, Germany) at 5,000 cells per well overnight. For PKH26‐labeled EV uptake, growth media was then replaced with addition of suspension of 5 × 10^9^ particles/ml for 18‐h. For Hrase I/III‐treated EVs, 1 mIU/ml of Hrase I (Sigma) and Hrase III (Sigma) were added to PKH26‐labeled EVs for 3‐h at 37°C. To remove Hrase I/III, EVs were re‐isolated by ultracentrifugation at 110,000*g* for 1 h. For Hrase I/III‐treated cells, 1 mIU/ml of Hrase I, III were added to cells for 3‐h at 37°C and fresh 1 mIU/ml of Hrase I, III were further added for 2‐h at 37°C. To avoid the recovery of intact cellular HSPGs, EVs were added for 4‐h. For immuno‐fluorescence, cells were fixed with 4% paraformaldehyde and permeabilized with 0.1% tween‐20 in PBS for 10 min at room temperature. The cells were blocked and incubated with fluorophore‐conjugated antibodies as indicated, or Phalloidin‐FITC (Sigma, P5258) for 2 h, whereas the nuclei were labelled with Nucblue DNA binding dye (Thermo Fisher Scientific, R37605). Images were collected using LSM780 confocal microscope (Carl Zeiss, Thornwood, NY,USA) with the 63×/1.40 or 40×/1.40 objective.

### 2.7 Confocal microscopy with fluorescent organelle tracker

Cells were plated on μ‐Slide 8‐Well ibiTreat chambered coverslips (ibidi, Germany) at a concentration of 5,000 cells per well overnight. To label the organelles, BODIPY FL Glibenclamide (Thermo Fisher Scientific, 5 μM) for ER, BODIPY‐FL‐ceramide (Thermo Fisher Scientific, 5 μM) for Golgi apparatus, or LysoTracker (Thermo Fisher Scientific, 5 μM) for lysosome were added to the cells for 30 min at 37°C. For EV uptake, the cells were washed once with PBS and twice with culture media. PKH26‐labeled EVs were added at a concentration of 5 × 10^9^ particles/ml for 18‐h. Images were collected using LSM780 confocal microscope (Carl Zeiss, Thornwood, NY) with the 63×/1.40.

### 2.8 Confocal spinning disk microscope for live cell EV tracking

For live cell imaging, RAS3 cells were treated with 1 × 10^10^ particles/ml of PKH26‐labeled EV preparation for 18‐h. Rapid time‐lapse imaging was performed using Zeiss spinning disk Axio Observer Z1 confocal microscope at 37°C within 5% CO_2_ environment. Live cell images were acquired every 5‐s using an Evolve 512 EMCCD camera with Alpha Plan‐Apo 63 × /1.46 objective. Cells were imaged for up to 5 min and particle tracking was performed using TrackMate for Fiji (Vesicle diameter = 1μm; thresholds and other parameters were chosen as appropriate based on control samples within each experiment).

### 2.9 SiRNA knockdown

Gene silencing was conducted using Accell SMARTpool siRNA for rat CRAF (E‐087699‐00‐0020, Thermo Scientific Dharmacon, Lafayette, CO, USA) (GCAGAGAGAUUCAAGUUAU, UCCUCAAUUAUGUUAUUUU, UCUUAAUGAUUUUGGGUUU, GUCACAUCCUUGUCU‐GUAA) or Accell Non‐targeting Pool for control (D‐001910‐10‐20, Thermo Scientific Dharmacon). For read‐out, either 5,000 cells were plated per well of the 6‐well plate for flow cytometry or 1,000 cells were seeded per μ‐Slide 8‐Well ibiTreat chambered coverslip for confocal microscopy overnight. Growth media was then replaced with the Accell siRNA Delivery Media containing 1 μM siRNA. After treatment with siRNA for 48‐h, a concentration of 5 × 10^9^ particles/ml of PKH26‐labeled EV was used to conduct 18‐h cell exposure.

### 2.10 MTS assay

Cells were seeded in 10‐cm plates at 1 × 10^6^ cells per plate overnight. Growth media was then replaced with 10 ml of culture media containing 50 μM EIPA (or control) for 19 h. EIPA‐treated or control cells were then trypsinized and seeded in 96‐well plates at 5 × 10^3^ cells per well. After 2‐days of incubation, media were removed and replaced with 100 μl of fresh media supplemented with 20 μl of CellTiter 96 AQ_ueous_ One Solution MTS Reagent (Promega, Madison, WI, USA). Plates were incubated at 37°C for 1‐h in a humidified, 5% CO_2_ atmosphere. Absorbance was recorded at 490 nm using a 96‐well plate reader. Background absorbance of only media with MTS reagent were subtracted for comparison.

### 2.11 Soft agar colony formation assay

Cells were seeded in 12‐well plate at 40,000 cells per well in soft agar (base: 1% agar; top: 0.5% agar). Fresh media with or without EIPA (50 μM) was added every 3‐days. After 9 days, the multicellular colonies were stained by CellTiter 96® AQ_ueous_ One Solution MTS Reagent and counted using the microscope.

### 2.12 In vivo mouse metastasis assay

All in vivo experiments were performed according to the Animal Use Protocol (AUP) approved by the Institutional Animal Facility Care Committee and following Guidelines of the Canadian Council of Animal Care (CCAC), as described earlier (Meehan et al., 2013). Mice, 20–23‐week‐old, harbouring yellow fluorescent protein transgene on the background of severe combined immunodeficiency (SCID or YFP/SCID; Charles River, Saint‐Constant, QC, Canada or own colony, respectively) were injected with control and EIPA pre‐treated (19 h) RAS3 cells *via* tail vein (n = 8 for control and n = 9 for EIPA pre‐treated RAS3 cells) at 2 × 10^5^ cells per mouse in 0.1 ml volume of PBS. After 4 weeks, mice were sacrificed and lung tissues were extracted to enumerate the number of metastatic nodules. Additional biological replicate (n = 7) were conducted using NSG mice. Parallel experiments interrogated subcutaneous tumour growth, as indicated.

### 2.13 Data analysis

The results of the EV analysis have been submitted to, and obtained, EV TRACK ID ‐ EV210108. All results have been repeated independently at least twice. For all numerical data the plotted numbers represent mean ± SD and the respective *p* values were calculated using one‐tailed t‐test in Prism version 7.0a (GraphPad Software, San Diego, CA). *P* values were indicated by asterisks and considered to be statistically significant for *P *< 0.05.

## 3 RESULTS

### 3.1 Oncogenic RAS activates the EV uptake switch from endocytosis to macropinocytosis

**FIGURE 1:**
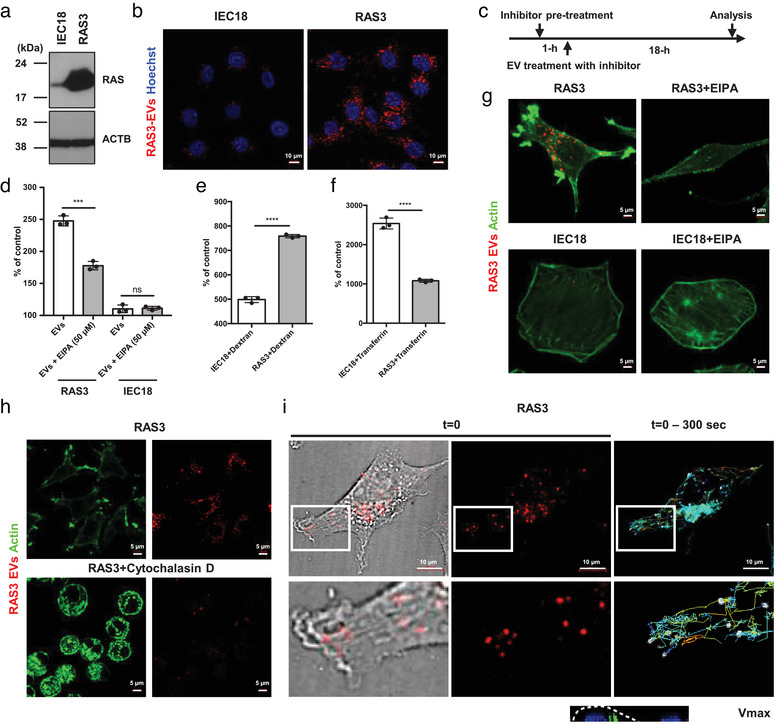
Mutant HRAS activates the uptake of EVs *via* macropinocytosis. (a) Western blotting illustrates the high level of HRAS proteins in RAS3 cells relative to their isogenic non‐transformed IEC18 counterparts. ACTB ‐ beta‐actin. (b) Confocal images show the increased uptake of PKH26‐labelled EVs by RAS3 cells compared to IEC18 cells. (c) Schema of EV and inhibitor treatment. (d) Flow cytometry analyses show the decreased uptake of PKH26‐labeld EVs by RAS3 cells co‐treated with EIPA relative to EV uptake by IEC18 cells, which was not affected. (e to f) Uptake of 70‐kDa dextran (e), known to enter cells through macropinocytosis (Commisso et al., 2013), was increased in RAS3 but they poorly internalized fluorescent transferrin (f), a substrate of endocytosis‐based cellular uptake pathway (Commisso et al., 2013), in flow cytometry. (g) RAS3 cells exhibit distinctive actin‐rich ruffle structures colocalized with sites of EV uptake, but these structures were absent after EIPA treatment, and in non‐transformed IEC18 cells. (h) Ruffle structures of RAS‐transformed cells were also disrupted following the inhibition of actin polymerization by cytochalasin D (Heusermann et al., 2016), resulting in the blockage of the EV entry into the cells. (i) Live cell imaging by spinning disk confocal microscope of RAS3 cells represents the active movement of EVs from their entry site at the membrane ruffle region to perinuclear regions where they subsequently accumulated

To glean more insights into reprogramming of EV communication mechanisms in cancer, we modelled RAS‐driven EV uptake in a series of isogenic cell lines including non‐transformed epithelial cells of intestinal (IEC18) or mammary origin (MCF10A) and their corresponding aggressive variants harbouring mutant HRAS (V12; RAS3 and MCF10AT, respectively) (Figure 1a, Fig. S2). We also compared isogenic human colorectal cancer cells with intact (DLD1, DKO1) or disrupted (DKS8) KRAS oncogene (Figure S2). Notably, the exposure to fluorescently labelled small exosome‐like EVs from RAS3 cells, or from other sources, led to limited vesicle internalization by indolent cells, a property that increased dramatically in the context of mutant RAS expression (Figure 1b, Fig. S2C to E).

Such uptake of EVs by cancer cells has been previously linked to processes of endocytosis (Christianson et al., 2013) or macropinocytosis (Nakase et al., 2015), the latter involving formation of circular engulfing membrane structures dependent on sodium hydrogen exchangers (NHEs) (Commisso et al., 2013; Kim et al., 2018). To discern between these possibilities, non‐transformed and RAS‐transformed cells were pre‐treated with NHE inhibitor [5‐(N‐Ethyl‐N‐isopropyl)amiloride; EIPA] and exposed to EVs labelled with the lipid binding fluorescent dye (PKH26) for 18‐h (Figure 1c). Under these conditions EV uptake is maximal between 12‐h to 24‐h (Figure S2F). While EIPA strongly inhibited EV engulfment by RAS‐transformed cells the residual EV uptake by their indolent counterparts remained largely unaffected (Figure 1d). Moreover, cells exhibiting high levels of mutant RAS‐expression and transformation (RAS3) exhibited elevated uptake of 70‐kDa dextran, known to enter cells through macropinocytosis (Commisso et al., 2013), but they poorly internalised fluorescent transferrin, a substrate of endocytosis‐based cellular uptake (Commisso et al., 2013). In contrast, transferrin was readily taken up by isogenic non‐transformed IEC18 cells, which weakly ingested 70‐kDa dextran (Figure 1e, f).

It should be noted that in cells where RAS‐transformation was dependent on a single copy of mutant KRAS and accompanied by multiple other naturally acquired oncogenic alterations the 70‐kDa dextran uptake did not differentiate between cells harbouring mutant RAS (DKO1) and those in which this oncogene has been selectively disrupted (DKS8; Figure S2G, H). Cells driven by a single copy of mutant RAS also exhibited a weaker enhancement of the EV uptake, and less dramatic cellular transformation (Figure S2I) (Demory et al., 2013). This may suggest that high levels of RAS transformation involving both mutation and over‐expression of RAS may be required for macropinocytosis to become a dominant mechanism of the EV uptake. Collectively, these observations suggest that high transforming activity of RAS triggers a switch in cellular mechanisms of extracellular particle internalisation in favour of macropinocytosis that chiefly drives the uptake of exogenous EVs.

### 3.2 Formation of RAS‐driven membrane ruffles coincides with EV macropinocytosis

As expected, the high expression of oncogenic RAS led to dramatic changes in cell shape resembling previously described epithelial‐to‐mesenchymal transition (EMT) (Tauro et al., 2013). These changes included focal clustering of actin resulting in formation of actin‐rich ruffle structures (Joneson et al., 1996) at distinct sites of the plasma membrane, and interestingly, in proximity to sites of fluorescent EV entry. These structures were absent in RAS‐transformed cells pretreated with EIPA, which exerted little effect on the shape and actin cytoskeleton of non‐transformed (IEC18) cells (Figure 1g).

Ruffle structures of RAS‐transformed cells were also disrupted following global inhibition of actin polymerization in the presence of cytochalasin D (Heusermann et al., 2016), similarly resulting in the blockage of EV entry into the cells (Figure 1h, Figure S3). Moreover, live cell imaging of RAS‐transformed cells revealed active movement of EVs from their entry point at the membrane ruffle region to perinuclear sites where they subsequently accumulated (Figure 1i, Movie S1). Indeed, life microscopy of RAS3 cells treated with EVs also suggest their engulfment in the ruffle region (Movie S2). Thus, while the requirement for RAS‐driven ruffle formation in the process of EV uptake still needs to be conclusively documented these membrane structures and the associated cytoskeleton appear to be proximal to the sites of EV engulfment.

### 3.3 EV internalization by RAS‐transformed cells depends on surface fibronectin and proteoglycan

**FIGURE 2:**
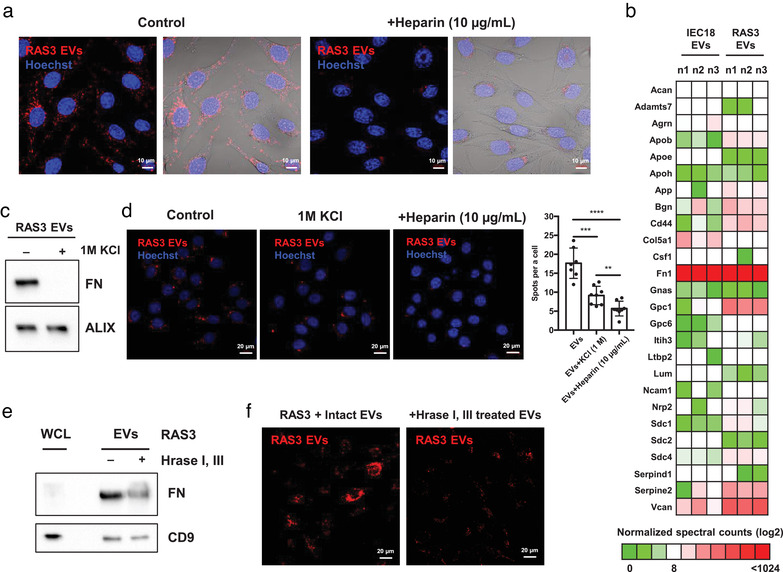
RAS‐driven EV internalization involves surface fibronectin‐HSPG interactions. (a) Confocal images (field of a 63× objective lens) show the decreased EV uptake following heparin co‐treatment of RAS3 cells. (b) Proteomes of IEC18 EVs and RAS3 EVs (Chennakrishnaiah et al., 2020) (three biological replicates) highlight the expression of HSPGs and HSPG binding proteins that could play a role in EV‐cellular interactions (see the GO analyses of proteomes in Figure S1). (c) High salt washing by 1 M KCl results in a complete disappearance of FN without affecting the levels of intravesicular marker ALIX. (d) Confocal images (field of a 40× objective lens) show the decreased uptake of KCl‐treated EVs by RAS3 cells. Internalized PKH26‐labeled EVs in confocal images were quantified by the total number of PKH26‐positive particle spots per cell (likely representative of clusters containing multiple EVs). Counting was conducted in 7 or 8 randomly taken images of individual cells. (e) Western blotting shows that Hrase I/III (heparinase) treatment removed the FN from EVs without affecting CD9 levels. (f) Confocal images (field of a 40× objective lens) show the decreased uptake of Hrase I/III‐pre‐treated EVs by RAS3 cells. *P* values (here and elsewhere: **** < 0.0001; *** < 0.001; ** < 0.01)

It is unclear whether macropinocytosis triggered by oncogenic RAS in cancer cells depends on specific molecular interactions at the cellular plasma membrane, or is largely unspecific (Commisso et al., 2013). It is noteworthy that initial molecular recognition events have been implicated in the EV‐cell contact prior to uptake (Basagiannis et al., 2016; Yao et al., 2019), including the requirement for the expression of heparin sulfate proteoglycan (HSPG) by EV recipient cells (Christianson et al., 2013; Purushothaman et al., 2016), or for interactions with integrin receptors (Hoshino et al., 2015). To explore these questions, RAS‐driven cells were exposed to fluorescent EVs in the presence of heparin, to block their contact with cellular HSPG (Christianson et al., 2013), which resulted in a significant inhibition of EV internalization (Figure 2a). As expected, the uptake of 70‐kDa dextran or transferrin were not affected by heparin (Figure S4A) indicting limitations of these tracers to mimic EV engulfment.

Since RAS‐transformed cells took up EVs from various cellular sources including both transformed (RAS3) and non‐transformed (IEC18) cells (Figure S1A), we mined the proteomes of RAS3 and IEC18 EVs for common HSPG binding proteins that could play a role in EV interactions with cellular HSPGs (Figure 2a, b, Figure S1A). Among several differentially expressed HSPG binding candidates (GPC1, CD44, SDC4), one common feature of both EV populations was the preponderance of EV‐associated fibronectin (FN). FN is known to associate with the surface of tumour‐derived EVs (Sung et al., 2015) and possesses the ability to interact with HSPG at the cellular plasma membrane (Heremans et al., 1990).

To assess the contribution of FN to RAS‐dependent vesicle internalization, EVs were stripped of the surface‐associated proteins in the presence of 1 M KCl (Figure 2c) resulting in the loss of FN signal, and subsequently incubated with RAS‐driven (RAS3) recipient cells (Figure 2d). While this treatment did not impact EV integrity (Figure S4B), it markedly reduced their cellular uptake, to an extent similar to that obtained in the presence of heparin (Figure 2d). This decreased uptake of protein stripped EVs was partially recovered by addition of exogenous FN (Figure S4C). In addition, when untreated EVs were incubated with target cells in the presence of the anti‐FN antibody their uptake was impaired (Figure S4D). These results suggest that FN present on the EV surfaces (perhaps other proteins as well) contributes to the elevated EV uptake by RAS‐transformed cells.

We observed that FN content was enriched in EVs relative to cell lysates and could be removed by KCl suggesting a preferential association of this protein with EV outer membranes. This association was also sensitive to treatment with heparinase I and III (Hrase I, III), which reduced the EV uptake by recipient RAS‐transformed cells (Figure 2e, f). Similarly, disruption of cellular HSPG by Hrase I, III in recipient cells induced the decreased EV uptake (Figure S4E) in keeping with prior reports (Christianson et al., 2013). In contrast, addition of RGD or HYD‐1 peptides blocking putative integrin receptors for FN had no effect on the EV uptake by RAS‐transformed cells (Figure S4F, G). Collectively, these observations suggest that the efficient RAS‐driven macropinocytosis of EVs depends on the exposure of HSPG on both cellular and EV surfaces and on surface FN. We propose that formation of HSPG‐FN‐HSPG bridges between EVs and recipient cells may occur prior to macropinocytotic EV internalisation (Figure S4H).

### 3.4 CRAF activity is required and sufficient for EV macropinocytosis

To understand molecular events driving EV macropinocytsis downstream of mutant RAS, cancer cells were pre‐treated with a series of pharmacological inhibitors prior to EV uptake (Figure S5 and Table S2). Somewhat surprisingly, inhibitors of pathways previously implicated in membrane interactions, EV uptake or macropinocytosis, such as MEK/MAPK (Trametinib, Selumetinib, PD98059, SCH772984) (Christianson et al., 2013), PI3K (LY294002, Wortmannin) (Commisso et al., 2013) or RALA/B (RBC8) (Jiang et al., 2016) had no effect on RAS‐driven EV internalization, as measured by flow cytometry. In contrast two different inhibitors of CRAF kinase activity (GW5074 and rocaglamide) significantly blocked RAS‐driven EV uptake (Figure S5). Moreover, confocal images revealed apparent colocalization of RAS, CRAF, and actin in ruffle region of RAS3 cells (Figure S6A), while in their non‐transformed (IEC18) counterparts the absence of ruffle structures coincided with the expression of endogenous wild type RAS (but not CRAF) near the plasma membrane (Figure S6B). Interestingly, macropinocytosis‐regulating NHE1 and NHE2 proteins were localized in proximity to CRAF within ruffle regions of RAS‐driven cancer cells (Figure S6C).

**FIGURE 3:**
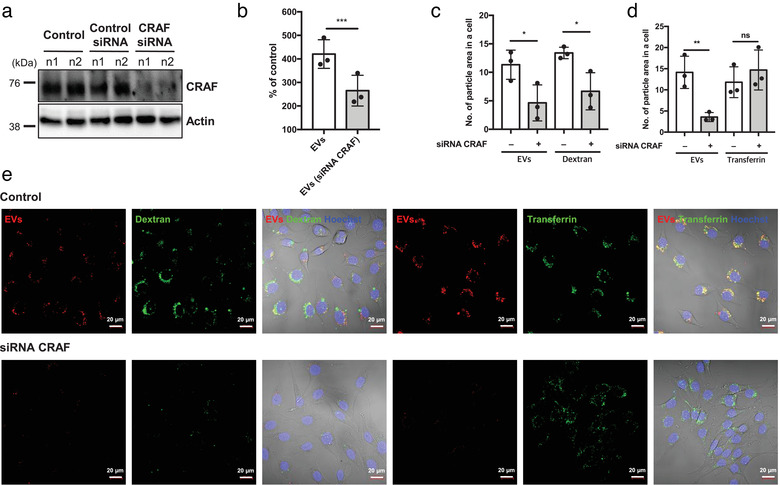
CRAF is required for particle macropinocytosis by HRAS transformed cells. (a) Western blotting documenting the downregulation of CRAF by siRNA in RAS3 cells. (b) Flow cytometry illustrates the reduced EV uptake by RAS3 cells following CRAF down‐regulation. (c to e) Confocal images documenting the decreased 70‐kDa dextran and EV uptake in CRAF siRNA‐treated RAS3 cells (c). However, transferrin uptake was not affected in CRAF siRNA treated RAS3 cells (d). Quantification of EV uptake in confocal images; fluorescent deposit counting was conducted in 3 randomly taken images of individual cells

The functional significance of these associations was further illustrated by a reduced fluorescent EV uptake in RAS‐driven cells in which CRAF was downregulated by siRNA (Figure 3a, b) along with decreased actin cytoskeleton clustering in ruffle regions (Figure S6D). These cells exhibited a comparable decrease in internalization of both 70‐kDa dextran and EVs, but not of fluorescent transferrin (Figure 3c‐e, Figure S7), findings consistent with the requirement for CRAF activation during macropinocytosis (but not endocytosis) driven by high levels of RAS activity.

**FIGURE 4:**
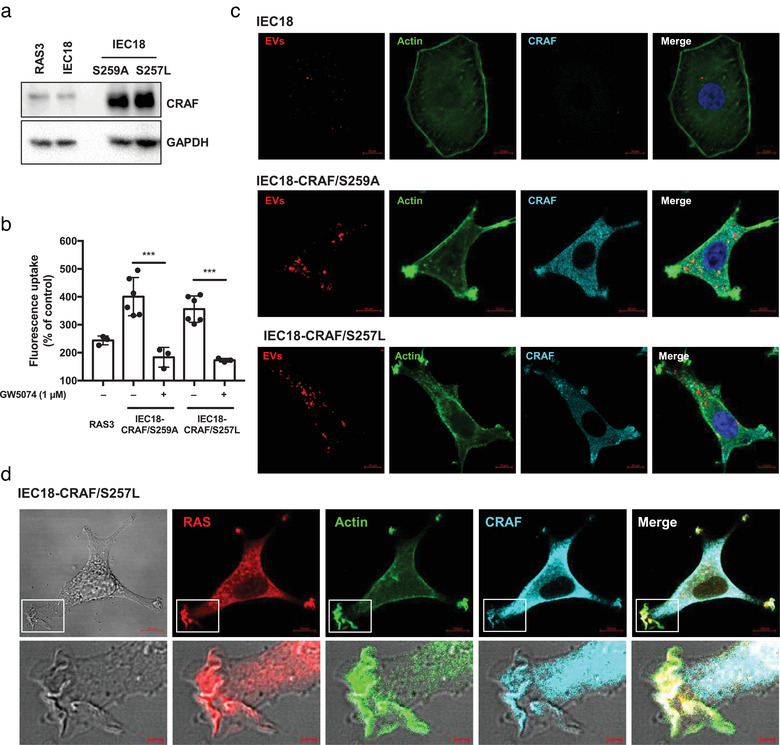
CRAF activity is sufficient to drive macropinocytosis. Overexpression of constitutively active mutants of the CRAF kinase (S259A or S257L) activates the EV uptake, causes phenotypic transformation and triggers formation of ruffle structures. (a) Western blotting documents overexpression of the CRAF kinase mutants in IEC18 cells including IEC18‐CRAF/S259A and IEC18‐CRAF/S257L cells. (b) Flow cytometry indicates the increased uptake of fluorescent RAS3 EVs by IEC18‐CRAF/S259A and IEC18‐CRAF/S257L relative to RAS3 cells. CRAF mediated EV uptake is obliterated by a specific CRAF inhibitor GW5407 (flow cytometry, three biological replicates for RAS3 and GW5074‐treated cells and six for control CRAF mutant IEC18 cells). (c) Confocal images illustrating a dramatic morphological transformation coupled with the generation of ruffle structures and increased uptake of fluorescent EVs by IEC18‐CRAF/S259A and IEC18‐CRAF/S257L cell lines compared to parental IEC18 cells. (d) Confocal images of immunofluorescent staining show that intracellular distribution of the endogenous RAS protein is re‐organized following activated CRAF expression. RAS signal was enhanced and clustered around ruffle region in contrast to its dimmer appearance and distribution around plasma membrane in non‐transformed cells

We next sought to assess whether activated CRAF is sufficient to drive macropinocytosis of exogenous EVs. To address this question non‐transformed IEC18 cells were engineered to overexpress two different constitutively active mutants of the CRAF kinase (S259A or S257L) (Dhillon et al., 2002), which resulted in a dramatic increase in fluorescent EV uptake (Figure 4a, b), an effect that could be reversed by CRAF kinase inhibitor (GW5074; Figure 4b). Notably, CRAF mutants also triggered changes in cell shape of IEC18 cells, which were reminiscent of RAS transformation and associated with formation of ruffle structures proximal to the site of intracellular accumulation of ingested EVs and enriched in CRAF, actin (Figure 4c) and RAS (Figure 4d). Interestingly, overexpression of wild type CRAF in IEC18 cells (IEC18‐CRAF/WT) also led to elevated EV uptake, cellular transformation and formation of ruffles but to a lesser extent than in the case of mutant CRAF (Figure S8). This may suggest that in the presence of high levels of CRAF the upstream events may lead to its activation.

### 3.5 Myosin phosphatase localizes with CRAF at sites of EV uptake

**FIGURE 5:**
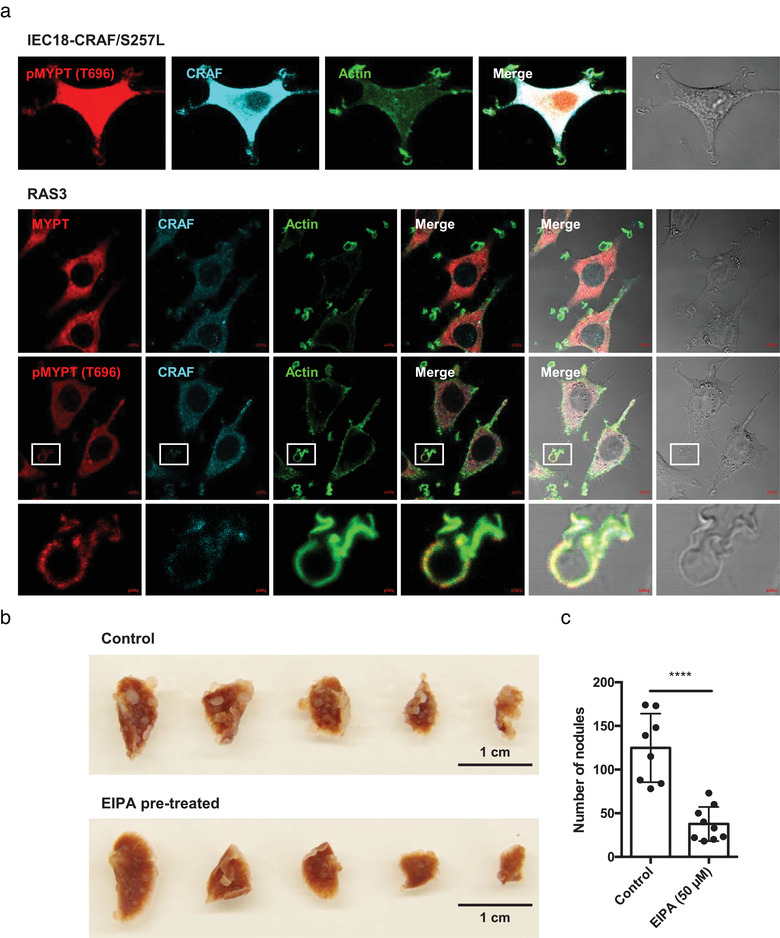
Signalling intermediates of the RAS/CRAF macropinocytosis pathway and its impact on experimental metastasis. (a) Phosphorylation of myosin phosphatase (MYPT) in cells harbouring transformed CRAF and RAS. Confocal images point to co‐localization between CRAF and pMYPT (T696) in IEC18‐CRAF/S257L and RAS3 cells. Additional co‐localization images were represented in Figure S9. (b and c) Metastasis assays: Control and EIPA pre‐treated (19 h) RAS3 cells were injected to the YFP‐SCID mouse *via* tail vein (n = 8 for control and n = 9 for EIPA pre‐treated RAS3 cells). After 4 weeks, mice were sacrificed and lung tissues were extracted to enumerate metastatic nodules. Additional biological replicate in NSG mice (n = 7) are presented in Figure S12C

Generation of crucial actin containing membrane ruffles involves myosin motor proteins (Cheresh et al., 1999) a process that entails RAF‐dependent phosphorylation of myosin phosphatase (MYPT) (Broustas et al., 2002) (Figure S7D). Indeed, our confocal images revealed a strong signal of both CRAF and phospho‐MYPT (T696) in actin‐rich ruffles of cells transformed with either mutant CRAF or HRAS (Figure 5a and Figure S9). Collectively, these results suggest that CRAF activation may represent a critical step in RAS‐driven ruffle formation and in the parallel enhancement of macropinocytosis of EVs by cancer cells.

### 3.6 Intracellular fate of engulfed EVs in RAS‐transformed cells

The intracellular fate of EVs and their cargo internalised by RAS‐transformed cells remains poorly studied. We observed that, in spite of different uptake mechanisms, both dextran and transferrin ultimately accumulate in perinuclear regions of the recipient RAS‐transformed cells (Figure S7E), a region also occupied by the bulk of internalised EVs. Confocal imaging and fluorescent tracker staining revealed that intracellular EV signal largely overlapped with the lysosome and partially also with endoplasmic reticulum (ER), but not with Golgi compartments (Figure S7F). Given this localization we reasoned that a subset of EVs transferred to the lysosome may undergo proteolytic degradation suggested to have a trophic effect on recipient cancer cells (Commisso et al., 2013). Indeed, GFP labelled EVs from A431 CD63/GFP cells (Montermini et al., 2015) were readily taken up by cells overexpressing mutant RAS and accumulated in the perinuclear region corresponding to lysosome. Within 24 h GFP protein signal (but not PKH26‐related lipid signal) was cleared from recipient cells suggesting degradation (Figure S10). This may suggest that following macropinocytosis EVs are rapidly degraded by recipient RAS‐driven cells.

### 3.7 RAS‐driven EV macropinocytosis is coupled with clonal growth and metastatic capacity

Internalisation and processing of EVs by RAS‐transformed cells would be expected to exert functional effects. While we were unable to detect mitogenic or migratory responses of RAS‐driven cells in relation to their enhanced EV uptake in vitro (Figure S11), interference with macropinocytosis did impact their clonogenic growth in vitro and in vivo. Thus, when RAS3 cells pretreated with the macropinocytosis inhibitor (EIPA) were injected intravenously into immune deficient YFP‐SCID mice the number of metastatic nodules was markedly reduced relative to untreated controls (Figure 5b, c), and without signs of overt toxicity to cells or animals (Figure S12). Moreover, intravenous injection RAS3 cells into more immunocompromised NSG mice lacking natural killer cell activity (Okada et al., 2019) resulted in expectedly higher number of metastases (Palumbo et al., 2005). Interestingly, this effect was markedly, albeit partially, attenuated when RAS3 cells were pre‐treated with EIPA prior to injection (Figure S12D). It is interesting to note that while EIPA reduced metastatic growth in both YFP‐SCID and NSG mice, the effects were more dramatic in the presence of NK cytotoxicity, which may point to the possible pro‐survival effect of macropinocytosis and the EV uptake in the face of innate immunity.

The partiality of EIPA effects on formation of metastatic lung nodules are understandable given the multifactorial nature of the tumour dissemination process (Fidler, 2003). It should also be kept in mind that EIPA effects are reversible as documented earlier with Madin‐Darby Canine Kidney (MDCK) cells in which the effects of this inhibitor lasted up to 2 h following its removal (Lagana et al., 2000). However, the fate of metastatic cells in the circulation (survival, arrest, extravasation) is often determined within minutes to hours post their vascular entry (Aslakson et al., 1991). Thus, if EIPA conferred even a short impairment of metastatic proficiency of RAS‐transformed cells their ability to form lung nodules would be expected to be reduced.

Our observations suggest that EIPA interferes with the clonal growth of intravenously injected cancer cells in lungs, but not with subcutaneous formation of the tumour mass following local inoculation of large numbers of RAS‐driven cells (Figure S12). Notably, the latter process is often considered to be polyclonal and unrelated to cancer cell stemness (Singh et al., 2004). Whether the EIPA sensitivity of the metastatic (clonal) growth of RAS‐driven cells depends on the uptake of autologous or host‐related EVs remains unclear. To glean more insights in this regard we tested the effect of EIPA on the clonal, adhesion‐independent cellular growth in vitro, using a soft agar colony formation assay. In this setting the only source of EVs were the RAS3 cells themselves. Interestingly, in this setting clonogenicity of RAS3 cells was strongly inhibited by EIPA (Figure S12E). This observation suggests that macropinocytosis remains relevant to the clonogenic potential of RAS‐driven cancer cells even in the presence of their own (autocrine) EVs.

## 4 DISCUSSION

Our observations reveal a new role of the oncogenic RAS/CRAF pathway in reprogramming of cellular EV uptake mechanisms by cancer cells. Notably, our data suggest that while non‐transformed epithelial cells internalize EVs (and other substrates) through the process of endocytosis, as described earlier (Svensson et al., 2013) the high level of mutant RAS expression drives a switch in which endocytosis is suppressed, while the EV uptake through macropinocytosis assumes a dominant role. While we postulate that this switch is responsible for a dramatic increase in EV uptake efficiency it is possible that this change also affects other substrates of macropinocyrosis, such as extracellular fluid or particles (Commisso et al., 2013).

The mechanisms of EV internalization by RAS‐transformed cells may be modulated by the level of RAS activity (expression/mutation) and interfering influences of the cellular background. While all cell lines expressing mutant RAS exhibited elevated EV uptake relative to controls the extent of this change differed markedly. For example, high level of mutant RAS expression in RAS3 cells originating from normal intestinal epithelial cells (IEC18) drove a dramatic change in cellular phenotype resembling EMT, along with startling biological aggressiveness and elevated EV uptake inhibitable by macropinocytosis blockade with EIPA. These features were attenuated in DKO1 cells harbouring a single copy of mutant RAS along with multiple accompanying genetic alterations (Berg et al., 2017; Shirasawa et al., 1993). This observation suggests that the core pathway of RAS‐driven EV uptake operative in the simplified case of RAS3 cells, may undergo quantitative and qualitative modulations in various cancer settings.

RAS‐driven process of EV engulfment by cancer cells is accompanied by formation of distinct actin rich ruffle structures enriched for assemblies of the respective regulatory proteins (RAS, CRAF, NHE, pMYPT). Our data suggest trafficking of internalized EVs into the lysosome and ER along with coordinated changes in cell shape and RAS/CRAF distribution and responsive to treatment with pharmacological inhibitors. Importantly, CRAF appears to be both necessary and sufficient to activate a robust EV uptake switch and the onset of efficient macropinocytosis.

We observed that activated/mutated CRAF appears to drive a particularly robust EV uptake (exceeding that of RAS‐transformed cells), in association with changes in cell shape and formation of membrane ruffles. Whether CRAF activation in the absence of oncogenic RAS or in its presence activates the same or different chains of events leading to EV macropinocytosis remains to be investigated. Moreover, while we consistently observed a strong association between ruffle formation and the elevated EV engulfment capacity by cancer cells along with spatial proximity of these structures to cellular entry sites of EVs, the mechanistic links between these properties in RAS‐transformed cells require further study.

Activation of RAS‐driven macropinocytosis was postulated to serve as a mechanism of replenishment of the cancer cell pool of biosynthetic substrates through utilization of extracellular sources of proteins and amino acids, thereby driving cellular growth (Commisso et al., 2013). Yao et al., reported that aborted macropinocytosis achieved by the knockout of the HSPG receptor, syndecan‐1, may drive down the lung metastatic potential and engender less clonogenic properties of pancreatic cancer cells (Yao et al., 2019). This study did not evaluate, however, whether these processes involve/impact the uptake of EVs. In this regard we observed that deregulation of macropinocytosis pathway appears to have a role in the EV engulfment and exerts a more specific impact on clonogenic (rather than general) growth properties of cancer cells. Moreover, in our hands, the exposure of HSPG on the surface of both, EVs and their recipient cells is required for the efficient EV uptake by RAS‐transformed cells with possible involvement of protein bridges, involving FN and/or possibly other HSPG‐binding proteins.

Functionally, our observations raise a possibility that EV uptake through macropinocytosis may play a role in clonal growth potential of RAS‐transformed cancer cells. This is of interest as clonal growth potential defines tumour initiating capabilities (stemness) of single cancer cells, which are the major determinant of metastasis, tumour relapse and other key events in the natural history of several human cancers. While different roles of EV communication in the biology of cancer stem cells have been investigated (Nakano et al., 2015), the respective contribution macropinocytosis to the colonogenic phenotype is still poorly understood. Whether the impact of the EV engulfment entails a large‐scale re‐utilization of EV‐associated macromolecules, as described earlier (Commisso et al., 2013; Kim et al., 2018), or other effects remains to be fully elucidated. It could be speculated that the elevated expenditure of membranes during the exacerbated RAS‐driven cancer cell vesiculation (Lazar et al., 2018) may also result in demand for lipids and other molecular cargo available through the uptake, or reuptake of EVs either autologous or host cell related.

In the light of our observations, development of genetic models of EV biogenesis, release, molecular reprogramming and uptake in the context of specific cancer driver events emerges as a pressing priority. As discussed earlier, testing the role of EV uptake processes in the absence of endogenous EV production would enable gaining a greater understanding of the possible differences between the biological effects of different exogenous EV subpopulations. Since EV uptake processes are, at least in theory, amenable to genetic screens it is possible to create such systems in order to validate and more efficiently target biologically relevant aspects of the EV communication, including the process macropinocytosis.

In this regard, the enhanced macropinocytosis in cancer cells has been implicated as a mechanism of selective drug delivery to RAS‐driven tumour cells (Kamerkar et al., 2017). However, the state of the RAS/RAF cascade in recipient cells and the exposure of patients to pharmacological inhibitors of different steps involved in this pathway (e.g. heparinoids), including of macropinocytosis (amiloride) ought to be considered among possible modulators of such approaches, which are also critically dependent on endosomal escape of the therapeutic cargo.

Since in RAS‐driven cells a fraction of intracellular EV‐related fluorescence colocalizes with ER it is possible that subsets of EVs may be poised for preferential endosomal escape or re‐emission, either intact or upon modification and with altered biological activity. Such effects have been recently inferred from EV trafficking between cancer and stromal cells in the course of metastasis (Luga et al., 2012) and are of great interest.

As mentioned earlier, our observations suggest that the intact macropinocytosis pathway plays a notable role in the capacity of RAS‐driven cells to initiate stem cell‐like, clonal growth in three‐dimensional culture, or its equivalent during metastatic colonization. EV uptake may represent an importnat component of this phenotype. Thus, modulation of the ability of cancer cells to utilize the EV uptake pathway may pave the way to innovative anticancer therapies targeting cancer cell stemness.

## CONFLICTS OF INTEREST

The authors report no conflicts of interest.

## Supporting information

Supporting information.

Supporting information.

Supporting information.

Supporting information.

Supporting information.
