# Supplementary material for: Oncogenic RAS drives the CRAF‐dependent extracellular vesicle uptake mechanism coupled with metastasis
Source: J Extracell Vesicles. 2021 Jun 10;10(8):e12091. doi: 10.1002/jev2.12091 (PMC8191585; doi:10.1002/jev2.12091)
Supplement: Supplementary file 1 — Supporting information. [file JEV2-10-e12091-s002.docx]

**Figure S1.** Proteomic composition of IEC18 and RAS3 EVs. RAS3 cells are derived from IEC18 cells through enforced expression of mutant V12-HRAS. (A) Flow cytometry shows the IEC18 EV and RAS3 EV uptake in RAS3 cells. (B) Proteomes of IEC18 and RAS3 EVs were re-analysed from previous published datasets[1], which include the three biologically replicated datasets of each proteome. Venn diagram shows the changed EV protein composition in RAS3 EVs including 145 proteins which are not identified in IEC18 EVs (Table S1). Label-free quantitation by the normalized total TIC in Scaffold software (<http://www.proteomesoftware.com>). Among identified proteins, total 180 proteins were significantly (*p* value < 0.05) deregulated by mutant HRAS overexpression including, 129 up-regulated proteins and 51 of down-regulated proteins in RAS3 EVs. (C-E) GO analyses of significantly affected 180 proteins shows the differently regulated categories in GO Cellular Components (C), Biological Processes (D), and Molecular Functions (E).

**Figure S2.** Increased EV uptake driven by oncogenic HRAS and KRAS mutations. (A) Western blotting of RAS3 cells and EVs shows the enriched canonical vesicular protein ALIX and CD9, while ACTB is depleted in the isolated EVs. (B) NTA shows the size distribution of isolated RAS3 EVs with 152.4 ± 0.8 nm of average size. (C) Flow cytometry shows the increased EV uptake in mutant V12HRAS overexpressing human breast epithelial (MCF10AT) cells relative to their isogenic parental MCF10A cells (three biological replicates). (D) Isogenic DLD1 (heterozygous mutant and wildtype KRAS expression) and DKO1 (homozygous expression of mutant KRAS) cells show the increased RAS3 EV uptake relative to their counterparts expressing only wild type KRAS (DKS8 cells; three biological replicates). (E) Confocal images illustrate the increased EV uptake in MCF10AT cells compared to MCF10A. (F) Flow cytometry shows EV uptake at 3-h, 6-h, 12-h, and 24-h. (G) 70 kDa dextran-Oregon Green® 488 were added for 18-h to the indicated cells with or without 50 µM of EIPA (for 1-h before dextran addition). Flow cytometry shows similar level of 70-kDa dextran uptake in both DKO1 and DKS8 cells (three biological replicates). (H) Confocal images illustrate the cellular shape differences between DKS8 and DKO1. Arrow indicates the ruffle structure. *P* values (here and elsewhere: **** < 0.0001; *** < 0.001; ** < 0.01; * < 0.05); (I) Inducible mutant HRAS expression in IEC18 cells modifies the EV uptake. Clone 25 is derived from IEC18 cells through expression of dexamethasone (Dex) inducible mutant HRAS. Western blotting (left panel) shows that 1 µM of Dex treatment in normal growth media stimulated a modest expression of HRAS protein in Clone 25 cells. Flow cytometry shows the increased PKH26-labeled RAS3 EV uptake in 1 µM of Dex treated Clone 25 cells (Clone 25-Dex).

**Figure S3.** Increased EV uptake by RAS-transformed cells depends on intact actin cytoskeleton. (A) RAS3 cells show a more robust uptake of GFP-tagged EVs from unrelated A431-CD63/GFP cell line than parental IEC18 cells. (B) Treatment with cytochalasin D, actin polymerization inhibitor, induced a disruption of the ruffle structure associated with the blockage of the EV entry into the cells.

**Figure S4.** RAS-driven uptake of EVs following modification of their surface proteins. (A) Flow cytometry shows that heparin treatment does not affect the uptake of 70-kDa dextran or transferrin. (B) NTA shows the similar size distribution of control and 1 M KCl-treated RAS3 EVs, implying that this treatment does not impact the EV integrity. (C) Confocal images (field captured with a 63× objective lens) show the recovered uptake by RAS3 cells of KCl-treated EVs co-treated with rat FN for 18-h. Rat FN was pre-incubated with EVs at 100 µg/mL in 30 µL at 37ºC for 30 min. (D) Confocal images of recipient RAS3 cells (field captured with a 63× objective lens) show their decreased EV uptake associated with co-treatment with anti-FN antibody for 18-h. Anti-FN antibody was pre-incubated with EVs at 2.4 ng/mL in 30 µL at 37ºC for 30 min. (E) Confocal images (captured with 63× objective lens) show the decreased uptake of EVs following pre-treatment of recipient RAS3 cells with Hrase I, III for 4 hours of EV treatment. Counting was conducted in 5 to 12 randomly taken images of individual cells. (F) Flow cytometry illustrates that the synthetic RGD peptide, which blocks the FN integrin receptors, did not block the EV uptake, but heparin inhibited the EV uptake by RAS3 cells. (G) Flow cytometry documents that HYD-1 peptide, which blocks laminin integrin receptors interactions, did not block the EV uptake (three biological replicates). (H) The model of the formation of HSPG-FN-HSPG bridges between EVs and recipient cells prior to macropinocytotic internalisation.

**Figure S5.** The effects of RAS downstream signaling inhibitors on the EV uptake by cancer cells. (A) Schema of EV and inhibitor treatment. (B) Representative histograms of flow cytometry profiles illustrating the effects of indicated signalling inhibitors on the uptake of fluorescent EVs by RAS3 cells. Inhibitors targeted CRAF (GW5074, rocaglamide), MEK1/2 (Trametinib, Selumetinib, PD98059), ERK1/2 (SCH772984), PI3K (LY264002, Wortmannin), and RALA/B (RBC8). Experiments were conducted with 3 – 5 biological replicates, as summarized in (C). (D) Western blotting illustrating the inhibitory effects of Trametinib and GW5074 on their targets (MEK, CRAF) as measured by phosphorylation of the respective substrates (ERK and MEK) in RAS3 cells.

**Figure S6.** Formation of ruffle structures in RAS-driven cells revealed by multicolor immunofluorescent staining. (A) Confocal images show the intracellular proximity of RAS, CRAF, and actin in ruffle region of RAS3 cells. (B) IEC18 cells show well-structured actin stress fibers with localization of only RAS, but not CRAF, near the plasma membrane. (C, D) Confocal images show that the macropinocytosis-regulating proteins, NHE1 (C) and NHE2 (D), are localized in the same microregions of RAS3 cells as CRAF, and within ruffle structures. (D) Confocal images show disrupted actin cytoskeleton clustering within ruffle regions following downregulation of CRAF by siRNA in RAS3 cells.

**Figure S7.** RAS-driven EV uptake by macropinocytsis is regulated by CRAF. Flow cytometry analyses show that the uptake of RAS3 EVs (A) and 70-kDa dextran (DEX) (B) is decreased, but the uptake of transferrin (TRF) (C) remained unaffected in RAS3 cells after silencing CRAF by treatment with siRNA. (D) The emerging model of RAS-driven molecular interactions during macropinocytosis of EVs by cancer cells. (E) Confocal images of subcellular localization of test probes (fluorescent 70-Dka dextran or transferrin) or EVs following their uptake by RAS3 cells. While dextran and transferrin entered cells through different mechanisms, they eventually accumulated in the perinuclear region. This region was also occupied by internalized fluorescent EVs suggesting a convergence of intracellular trafficking pathways of exogenous material. (F) Staining for fluorescent organelle trackers reveals that RAS-driven cells concentrate ingested EVs in the lysosome, and to a lesser extent in ER, but not in the Golgi network (confocal microscopy 18 hours post EV exposure).

**Figure S8.** Confocal images illustrating a morphological transformation in IEC18 cells expressing wild type CRAF. While less pronounced that in the case of activating CRAF mutations CRAF overexpressing cells (IEC18-CRAF/WT) exhibit reorganization of actin cytoskeleton and increased uptake of fluorescent EVs.

**Figure S9.** Confocal images of co-localization between CRAF and pMYPT (T696) in IEC18-CRAF/S257L and RAS3 cells.

**Figure S10.** Transient intracellular retention of EVs following their uptake by RAS-driven cells. Confocal images show that in RAS3 cells the intracellular green fluorescent (GFP – top panels) signal resulting from the uptake of EVs generated by A431-CD63/GFP cells disappears in less than 2 days post EV addition. In contrast, red fluorescent signal resulting from exposure to lipid labelled EVs (PKH26) remains detectable after 4 days. Whether this is a result of processing EV lipids or tracer turnover remains uncertain.

**Figure S11.** Impact of EVs on cancer cell growth and migration *in vitro*. (A) Growth of RAS3 cells in standard monolayer culture in the presence or absence of the RAS3 EVs with 5 × 10^9^ particles/mL in serum-free media for 48-h and 72-h. Cell proliferation was tested using the metabolic activity readout through MTS assay. No significant effect of RAS3 EVs on the proliferation/survival of RAS3 cells was detected. (B) Migration of RAS3 cells were measured by wound healing assay. RAS3 cells were monitored presence or absence of the RAS3 EVs with 5 × 10^9^ particles/mL in serum-free media for 48-h. Changes in migration of RAS3 cells treated with EVs were not observed.

**Figure S12.** Impact of macropinocytosis inhibition on cancer cell growth *in vitro* and *in vivo*. (A) Growth of RAS3 cells in standard monolayer culture in the presence or absence of the macropinocytosis inhibitor, EIPA. IEC18 and RAS3 cells treated with control media or EIPA (for 19 hours) were cultured with normal culture media for 2-days and tested for metabolic activity using MTS assay. No significant effect of EIPA on the proliferation/survival of either IEC18 or RAS3 cells was detected. (B) Tumor growth in mice following injection of RAS3 cells revealed no impact of EIPA pretreatment. RAS3 cells (EIPA-RAS3) were injected at subcutaneously into immunodeficient YFP-SCID mice (n=5), at 2 × 10^5^ cells per animal. There was no significant difference of tumor weight after 17 days of growth. (C, D) A marked reduction in metastatic potential of RAS3 cells following EIPA pre-treatment (biological replicate of Fig. 5C) in YFP/SCID mouse (C) and NSG mouse (D). Control medium or EIPA containing medium were used to pre-treat RAS3 cells (for 19 hours) followed by intravenous injection into YFP/SCID mice (experimental metastasis model; n=7) and NSG mice (experimental metastasis model; n=7 for control and n=8 for EIPA pre-treated RAS3 cells). After 4 weeks, mice were sacrificed and lung tissues were extracted and enumerated for macroscopic tumor nodules. (E) Inhibition of clonogenic growth of RAS3 cells following EIPA treatment. Soft agar colony forming assay reveals a marked but not complete diminution of clonogenic anchorage independent potential in the presence of macropinocytosis inhibitor.

**Movies S1.** Live cell movie depicting intracellular EV movement. Extension of the experiment from Fig. 1I.

**Movies S2.** Live cell movie depicting extracellular EV.

**Table S2.** IC50 information for the used inhibitors.

| Name | Company | Catalog no. | MW | Target | IC50* | Used concentration in this study | Reference |
| --- | --- | --- | --- | --- | --- | --- | --- |
| Cytochalasin D | Sigma | C8273 | 507.6 | Actin | 0.5 µM [2] | 10 µM | [3] |
| EIPA | Sigma | A3085 | 299.76 | NHE1 | 0.02 µM [4, 5] | 50 µM | [5, 6] |
| GW5074 | Sigma | G6416 | 520.94 | CRAF | 9 nM | 1 µM | [7] |
| LY294002 | Selleckchem | S1105 | 307.34 | PI3K | 0.5 μM (PI3Kα); 0.57 μM (PI3Kδ); 0.97 μM (PI3Kβ) | 50 µM | [8] |
| PD98059 | Calbiochem | 513000 | 267.3 | MEK | 2 µM | 20 µM | [9] |
| RBC8 | Sigma | SML1295 | 424.45 | RalA/B | 2 µM | 50 µM | [10] |
| Rocaglamide A | Sigma | SML0656 | 505.56 | CRAF | 8.1 nM [11] | 200 nM | [11, 12] |
| SCH772984 | Selleckchem | S7101 | 587.67 | ERK1/2 | 4 nM (ERK1); 1 nM (ERK2) | 10 µM | [13] |
| Selumetinib | Selleckchem | S1008 | 457.68 | MEK1/2 | 14 nM (MEK1); 530 nM (MEK2) | 10 µM | [14] |
| Trametinib | Selleckchem | S2673 | 615.39 | MEK1/2 | 0.92 nM (MEK1); 1.8 nM (MEK2) | 10 µM | [15] |
| Wortmannin | Selleckchem | S2758 | 428.43 | PI3K | 3 nM | 500 nM | [8] |

*IC50 value is indicated from the datasheet of company or reference

**References**

[1] S. Chennakrishnaiah, T. Tsering, C. Gregory, N. Tawil, C. Spinelli, L. Montermini, N. Karatzas, S. Aprikian, D. Choi, L. Klewes, S. Mai, J. Rak, Extracellular vesicles from genetically unstable, oncogene-driven cancer cells trigger micronuclei formation in endothelial cells, Sci Rep 10(1) (2020) 8532.

[2] D. MacGlashan, Jr., N. Vilarino, Polymerization of actin does not regulate desensitization in human basophils, J Leukoc Biol 85(4) (2009) 627-37.

[3] T.M. Link, U. Park, B.M. Vonakis, D.M. Raben, M.J. Soloski, M.J. Caterina, TRPV2 has a pivotal role in macrophage particle binding and phagocytosis, Nat Immunol 11(3) (2010) 232-9.

[4] B. Masereel, L. Pochet, D. Laeckmann, An overview of inhibitors of Na(+)/H(+) exchanger, Eur J Med Chem 38(6) (2003) 547-54.

[5] A. Lagana, J. Vadnais, P.U. Le, T.N. Nguyen, R. Laprade, I.R. Nabi, J. Noel, Regulation of the formation of tumor cell pseudopodia by the Na(+)/H(+) exchanger NHE1, J Cell Sci 113 ( Pt 20) (2000) 3649-62.

[6] S. Taverna, M. Pucci, M. Giallombardo, M.A. Di Bella, M. Santarpia, P. Reclusa, I. Gil-Bazo, C. Rolfo, R. Alessandro, Amphiregulin contained in NSCLC-exosomes induces osteoclast differentiation through the activation of EGFR pathway, Sci Rep 7(1) (2017) 3170.

[7] P.C. Chin, L. Liu, B.E. Morrison, A. Siddiq, R.R. Ratan, T. Bottiglieri, S.R. D'Mello, The c-Raf inhibitor GW5074 provides neuroprotection in vitro and in an animal model of neurodegeneration through a MEK-ERK and Akt-independent mechanism, J Neurochem 90(3) (2004) 595-608.

[8] V. Chandramohan, S. Jeay, S. Pianetti, G.E. Sonenshein, Reciprocal control of Forkhead box O 3a and c-Myc via the phosphatidylinositol 3-kinase pathway coordinately regulates p27Kip1 levels, J Immunol 172(9) (2004) 5522-7.

[9] E.M. Wauson, M.L. Guerra, B. Barylko, J.P. Albanesi, M.H. Cobb, Off-target effects of MEK inhibitors, Biochemistry 52(31) (2013) 5164-6.

[10] C. Yan, D. Liu, L. Li, M.F. Wempe, S. Guin, M. Khanna, J. Meier, B. Hoffman, C. Owens, C.L. Wysoczynski, M.D. Nitz, W.E. Knabe, M. Ahmed, D.L. Brautigan, B.M. Paschal, M.A. Schwartz, D.N. Jones, D. Ross, S.O. Meroueh, D. Theodorescu, Discovery and characterization of small molecules that target the GTPase Ral, Nature 515(7527) (2014) 443-7.

[11] G. Polier, J. Neumann, F. Thuaud, N. Ribeiro, C. Gelhaus, H. Schmidt, M. Giaisi, R. Kohler, W.W. Muller, P. Proksch, M. Leippe, O. Janssen, L. Desaubry, P.H. Krammer, M. Li-Weber, The natural anticancer compounds rocaglamides inhibit the Raf-MEK-ERK pathway by targeting prohibitin 1 and 2, Chem Biol 19(9) (2012) 1093-104.

[12] B. Wang, Y. Li, F. Tan, Z. Xiao, Chinese herb derived-Rocaglamide A is a potent inhibitor of pancreatic cancer cells, Am J Transl Res 8(2) (2016) 1047-54.

[13] H. Lavoie, M. Sahmi, P. Maisonneuve, S.A. Marullo, N. Thevakumaran, T. Jin, I. Kurinov, F. Sicheri, M. Therrien, MEK drives BRAF activation through allosteric control of KSR proteins, Nature 554(7693) (2018) 549-553.

[14] C. Bartholomeusz, T. Oishi, H. Saso, U. Akar, P. Liu, K. Kondo, A. Kazansky, S. Krishnamurthy, J. Lee, F.J. Esteva, J. Kigawa, N.T. Ueno, MEK1/2 inhibitor selumetinib (AZD6244) inhibits growth of ovarian clear cell carcinoma in a PEA-15-dependent manner in a mouse xenograft model, Mol Cancer Ther 11(2) (2012) 360-9.

[15] J.G. Qiu, Y.J. Zhang, Y. Li, J.M. Zhao, W.J. Zhang, Q.W. Jiang, X.L. Mei, Y.Q. Xue, W.M. Qin, Y. Yang, D.W. Zheng, Y. Chen, M.N. Wei, Z. Shi, Trametinib modulates cancer multidrug resistance by targeting ABCB1 transporter, Oncotarget 6(17) (2015) 15494-509.
