# Supplementary material for: Oncogenic RAS drives the CRAF‐dependent extracellular vesicle uptake mechanism coupled with metastasis
Source: J Extracell Vesicles. 2021 Jun 10;10(8):e12091. doi: 10.1002/jev2.12091 (PMC8191585; doi:10.1002/jev2.12091)
Supplement: Supplementary file 5 — Supporting information. [file JEV2-10-e12091-s004.pptx]

## Slide 1
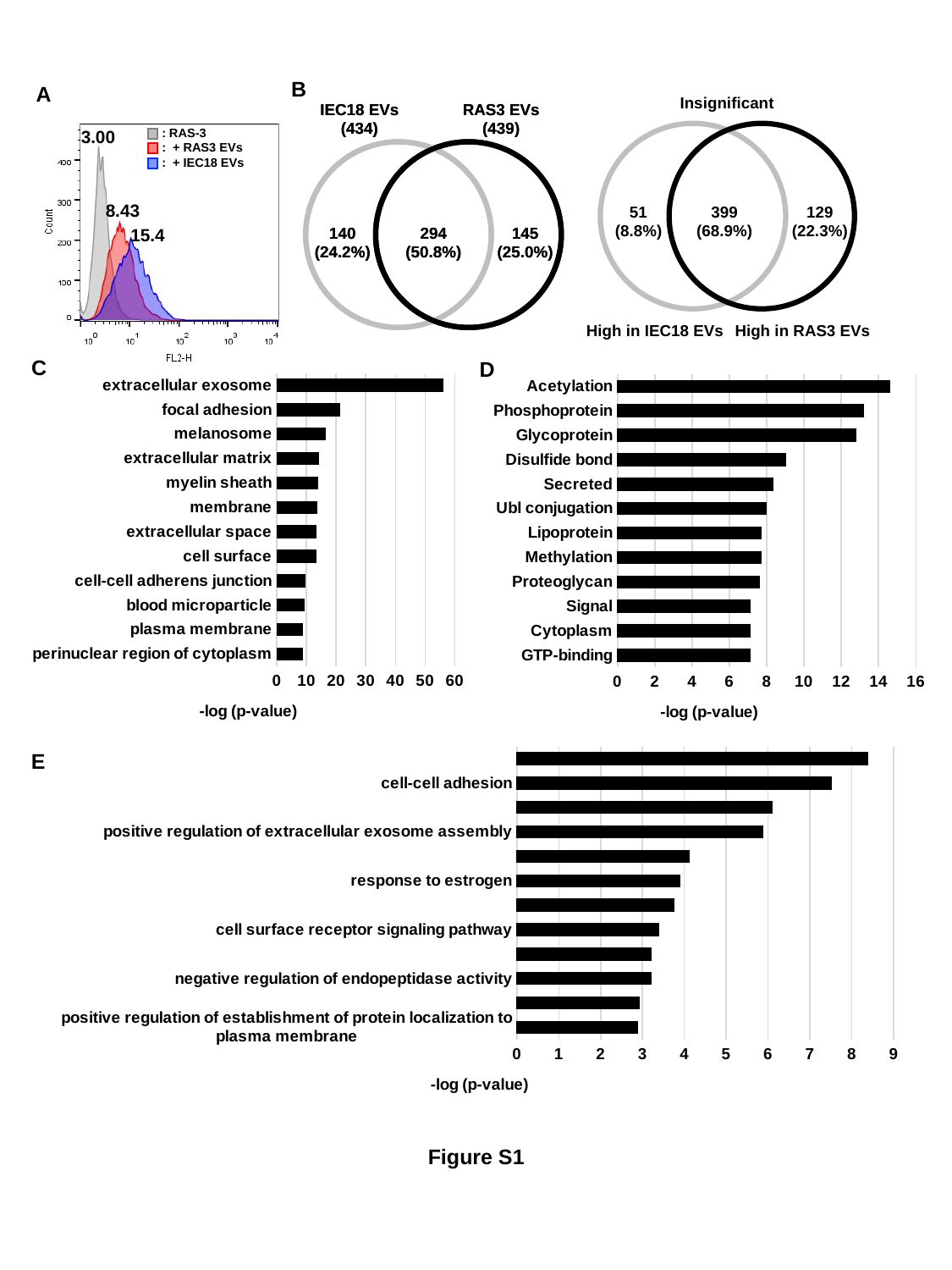

B
A
Insignificant
399
(68.9%)
51
(8.8%)
129
(22.3%)
High in IEC18 EVs
High in RAS3 EVs
IEC18 EVs
(434)
IEC18 EVs
(434)
RAS3 EVs
(439)
RAS3 EVs
(439)
: RAS-3
: + RAS3 EVs
: + IEC18 EVs
3.00
8.43
140
(24.2%)
140
(24.2%)
294
(50.8%)
294
(50.8%)
145
(25.0%)
145
(25.0%)
15.4
C
D
### Chart
| Category | |
|---|---|
| perinuclear region of cytoplasm | 8.657577319177793 |
| plasma membrane | 8.744727494896694 |
| blood microparticle | 9.318758762624412 |
| cell-cell adherens junction | 9.585026652029182 |
| cell surface | 13.27572413039921 |
| extracellular space | 13.3767507096021 |
| membrane | 13.48148606012211 |
| myelin sheath | 13.85387196432176 |
| extracellular matrix | 14.050609993355087 |
| melanosome | 16.42021640338319 |
| focal adhesion | 21.275724130399208 |
| extracellular exosome | 56.06550154875642 |
### Chart
| Category | |
|---|---|
| GTP-binding | 7.113509274827517 |
| Cytoplasm | 7.102372908709557 |
| Signal | 7.113509274827517 |
| Proteoglycan | 7.602059991327962 |
| Methylation | 7.721246399047171 |
| Lipoprotein | 7.698970004336018 |
| Ubl conjugation | 7.958607314841775 |
| Secreted | 8.346787486224654 |
| Disulfide bond | 9.0268721464003 |
| Glycoprotein | 12.795880017344075 |
| Phosphoprotein | 13.207608310501746 |
| Acetylation | 14.585026652029182 |
### Chart
| Category | |
|---|---|
| positive regulation of establishment of protein localization to plasma membrane | 2.886056647693163 |
| positive regulation of gene expression | 2.920818753952375 |
| negative regulation of endopeptidase activity | 3.2076083105017457 |
| positive regulation of cell migration | 3.214670164989233 |
| cell surface receptor signaling pathway | 3.387216143280264 |
| positive regulation of exosomal secretion | 3.7447274948966935 |
| response to estrogen | 3.886056647693163 |
| aging | 4.119186407719208 |
| positive regulation of extracellular exosome assembly | 5.886056647693162 |
| small GTPase mediated signal transduction | 6.107905397309518 |
| cell-cell adhesion | 7.522878745280337 |
| cell adhesion | 8.376750709602097 |E
Figure S1

## Slide 2
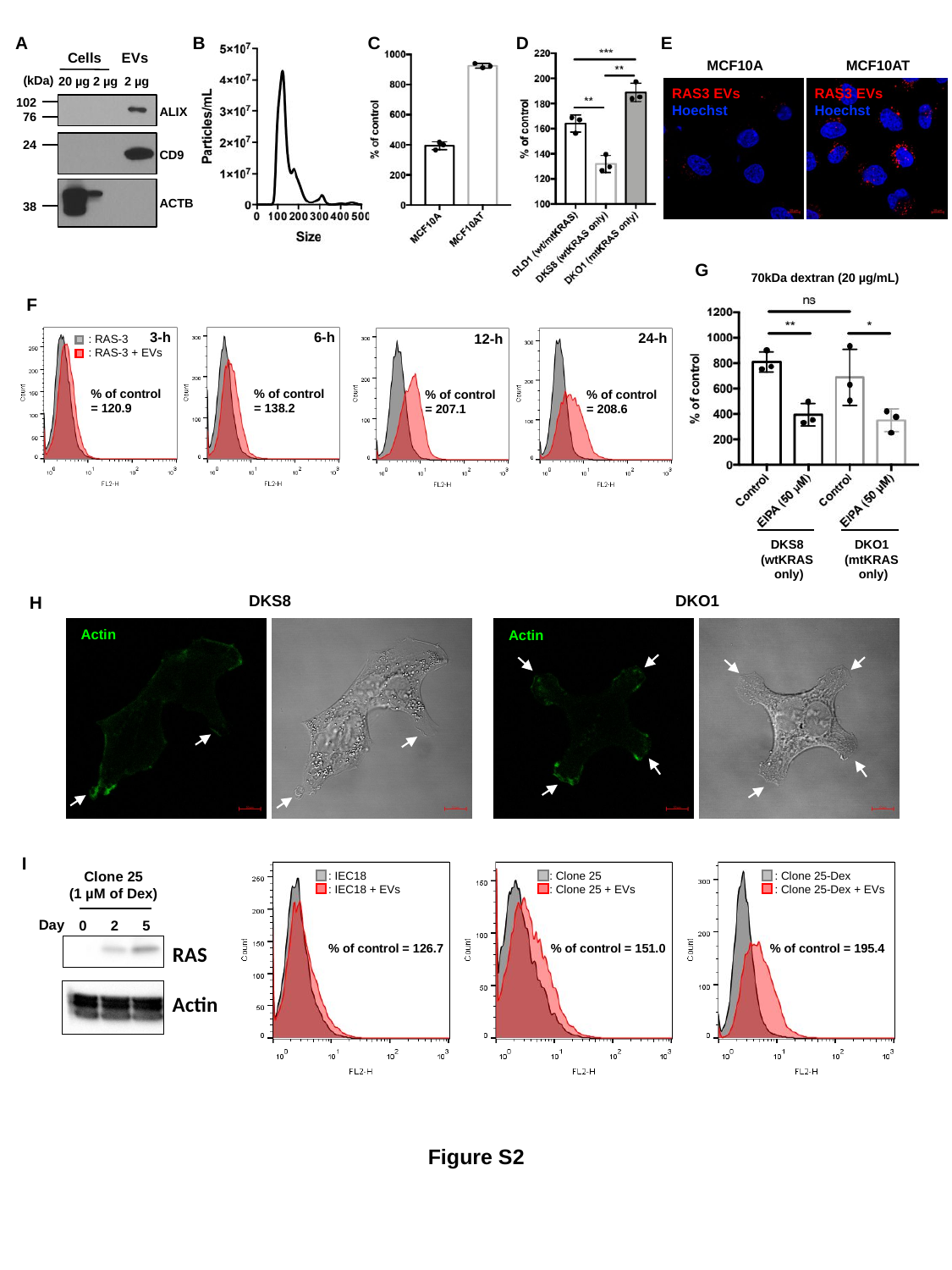

A
B
C
D
E
Cells
EVs
MCF10A
MCF10AT
(kDa)
20 µg 2 µg 2 µg
RAS3 EVs
Hoechst
RAS3 EVs
Hoechst
102
ALIX
76
24
CD9
ACTB
38
G
70kDa dextran (20 µg/mL)
DKS8
(wtKRAS
only)
DKO1
(mtKRAS
only)
F
3-h
6-h
: RAS-3
: RAS-3 + EVs
% of control
= 120.9
% of control
= 138.2
24-h
12-h
% of control
= 207.1
% of control
= 208.6
DKS8
DKO1
H
Actin
Actin
Clone 25
(1 µM of Dex)
: IEC18
: IEC18 + EVs
: Clone 25
: Clone 25 + EVs
: Clone 25-Dex
: Clone 25-Dex + EVs
Day
0
2
5
% of control = 126.7
% of control = 151.0
% of control = 195.4
RAS
Actin
I
Figure S2

## Slide 3
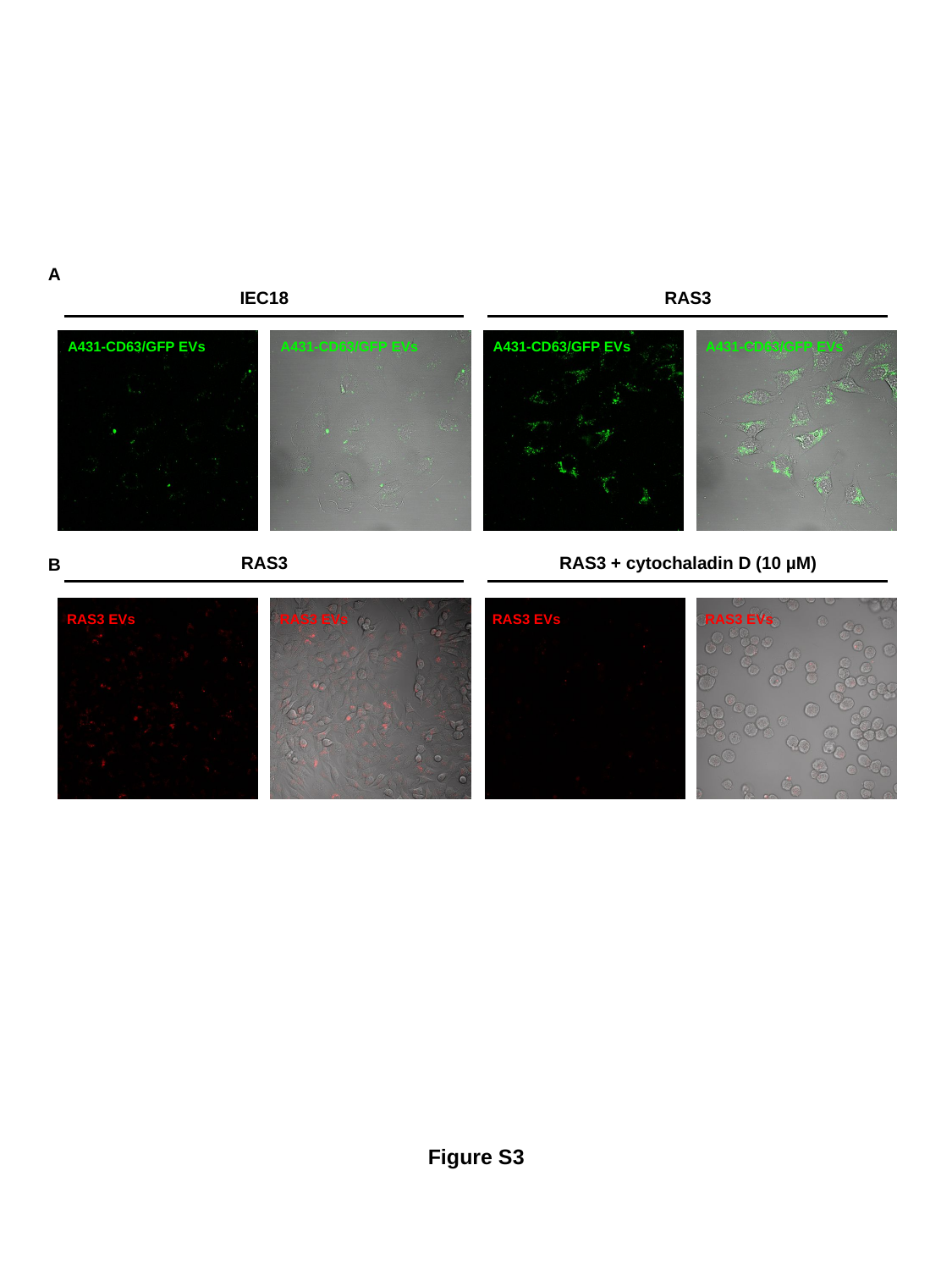

A
IEC18
RAS3
A431-CD63/GFP EVs
A431-CD63/GFP EVs
A431-CD63/GFP EVs
A431-CD63/GFP EVs
RAS3
RAS3 + cytochaladin D (10 µM)
B
RAS3 EVs
RAS3 EVs
RAS3 EVs
RAS3 EVs
Figure S3

## Slide 4
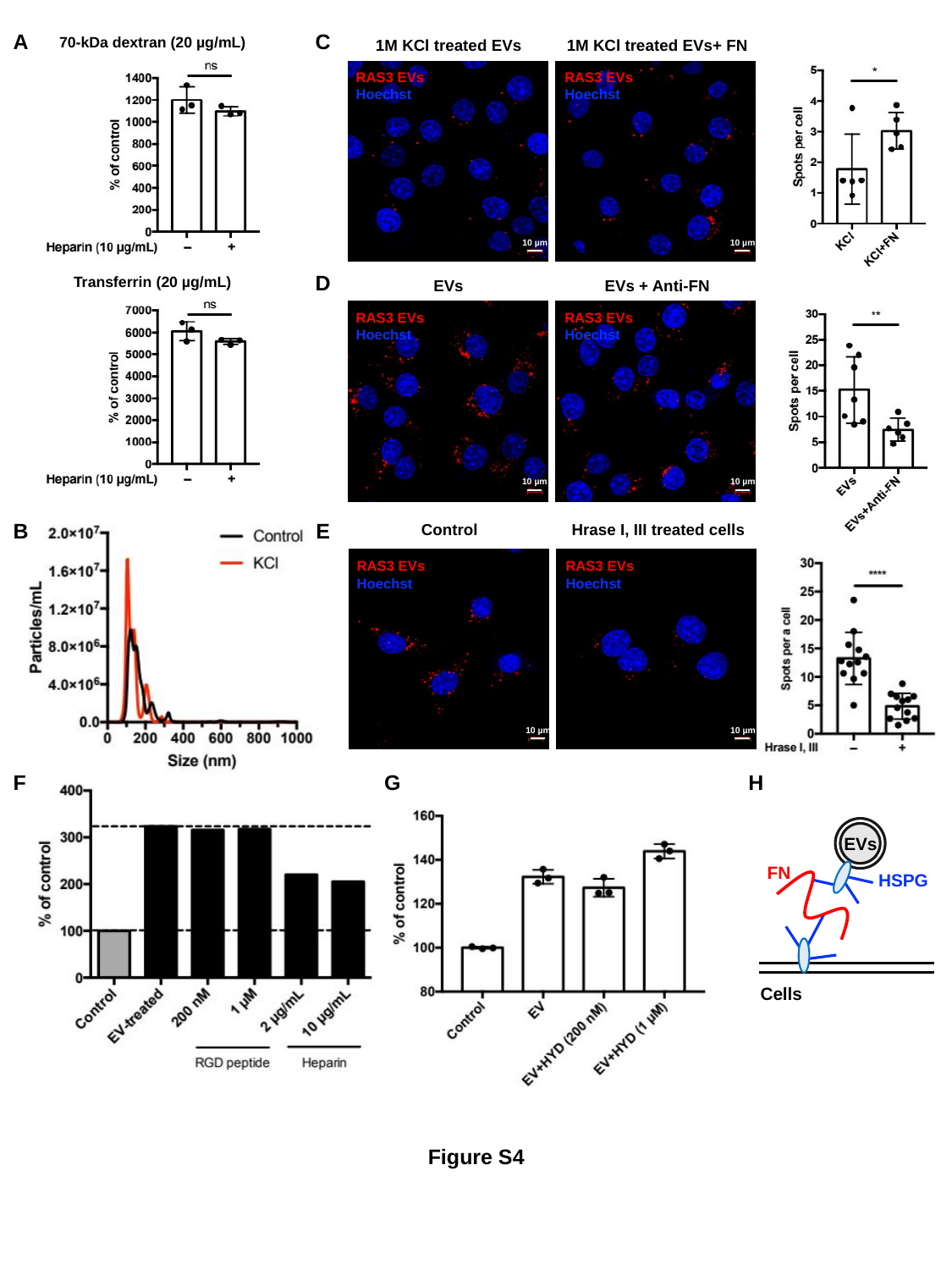

A
C
70-kDa dextran (20 µg/mL)
1M KCl treated EVs
1M KCl treated EVs+ FN
RAS3 EVs
Hoechst
RAS3 EVs
Hoechst
10 µm
10 µm
D
Transferrin (20 µg/mL)
EVs
EVs + Anti-FN
RAS3 EVs
Hoechst
RAS3 EVs
Hoechst
10 µm
10 µm
B
E
Control
Hrase I, III treated cells
RAS3 EVs
Hoechst
RAS3 EVs
Hoechst
10 µm
10 µm
F
G
H
EVs
FN
HSPG
Cells
Figure S4

## Slide 5
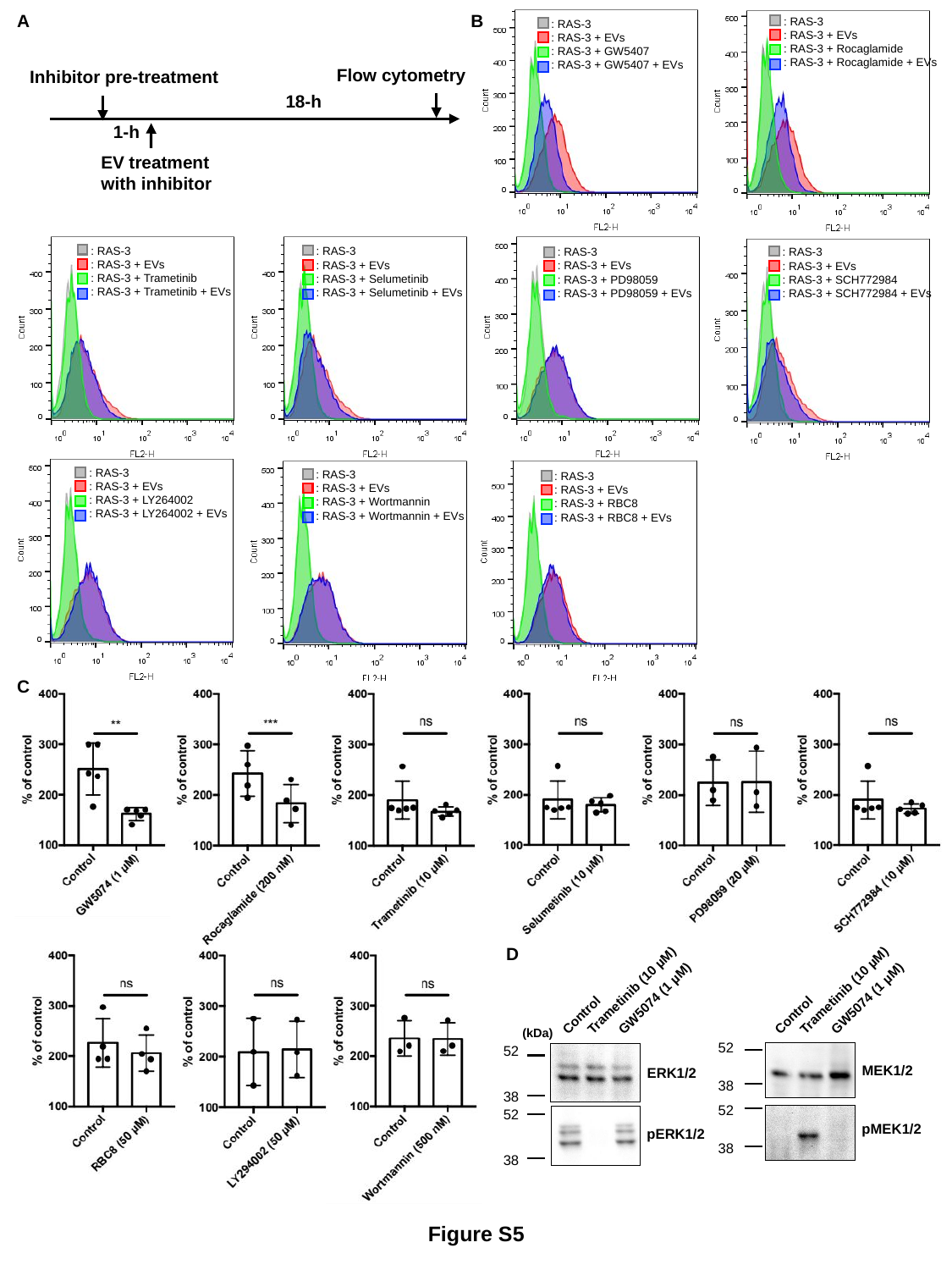

A
B
: RAS-3
: RAS-3 + EVs
: RAS-3 + Rocaglamide
: RAS-3 + Rocaglamide + EVs
: RAS-3
: RAS-3 + EVs
: RAS-3 + GW5407
: RAS-3 + GW5407 + EVs
Flow cytometry
Inhibitor pre-treatment
18-h
1-h
EV treatment
with inhibitor
: RAS-3
: RAS-3 + EVs
: RAS-3 + Trametinib
: RAS-3 + Trametinib + EVs
: RAS-3
: RAS-3 + EVs
: RAS-3 + Selumetinib
: RAS-3 + Selumetinib + EVs
: RAS-3
: RAS-3 + EVs
: RAS-3 + PD98059
: RAS-3 + PD98059 + EVs
: RAS-3
: RAS-3 + EVs
: RAS-3 + SCH772984
: RAS-3 + SCH772984 + EVs
: RAS-3
: RAS-3 + EVs
: RAS-3 + LY264002
: RAS-3 + LY264002 + EVs
: RAS-3
: RAS-3 + EVs
: RAS-3 + Wortmannin
: RAS-3 + Wortmannin + EVs
: RAS-3
: RAS-3 + EVs
: RAS-3 + RBC8
: RAS-3 + RBC8 + EVs
C
D
Trametinib (10 µM)
GW5074 (1 µM)
Control
Trametinib (10 µM)
GW5074 (1 µM)
Control
(kDa)
52
52
MEK1/2
ERK1/2
38
38
52
52
pMEK1/2
pERK1/2
38
38
Figure S5

## Slide 6
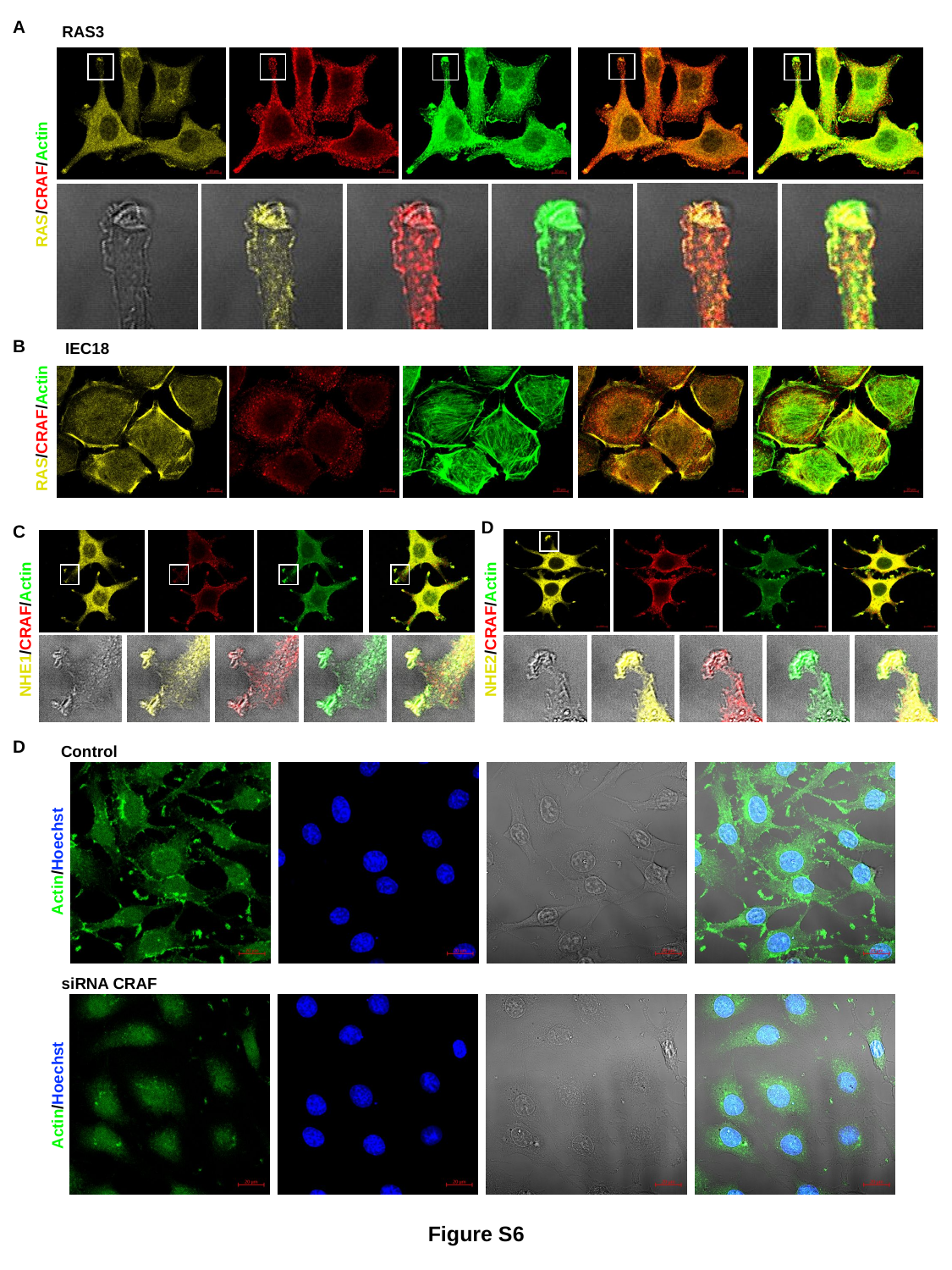

A
RAS3
RAS/CRAF/Actin
B
IEC18
RAS/CRAF/Actin
D
C
NHE1/CRAF/Actin
NHE2/CRAF/Actin
D
Control
Actin/Hoechst
siRNA CRAF
Actin/Hoechst
Figure S6

## Slide 7
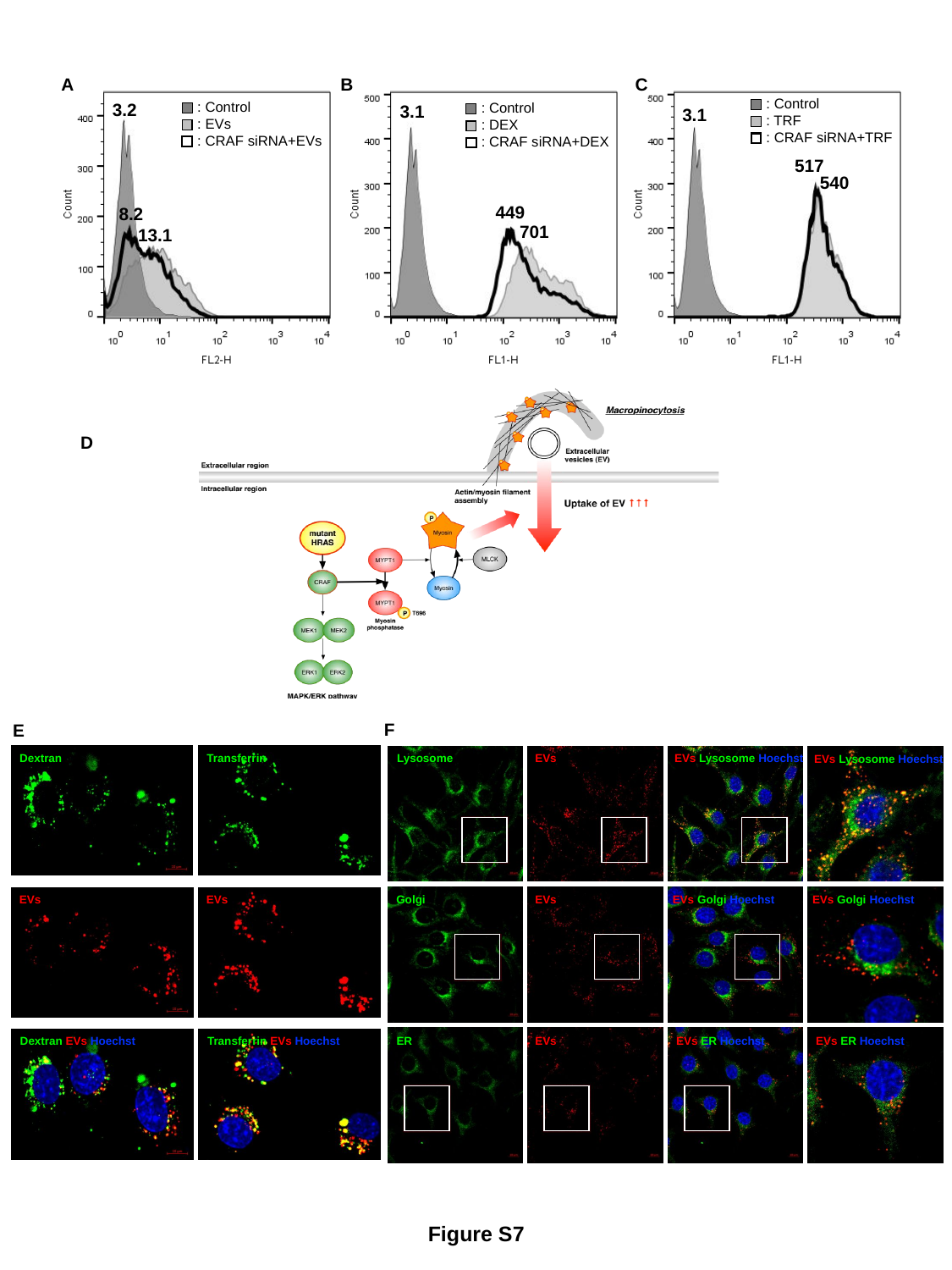

A
B
C
: Control
: TRF
: CRAF siRNA+TRF
3.2
: Control
: EVs
: CRAF siRNA+EVs
: Control
: DEX
: CRAF siRNA+DEX
3.1
3.1
517
540
449
8.2
701
13.1
D
F
E
Dextran
Transferrin
Lysosome
EVs
EVs Lysosome Hoechst
EVs Lysosome Hoechst
EVs
EVs
Golgi
EVs
EVs Golgi Hoechst
EVs Golgi Hoechst
Dextran EVs Hoechst
Transferrin EVs Hoechst
ER
EVs
EVs ER Hoechst
EVs ER Hoechst
Figure S7

## Slide 8
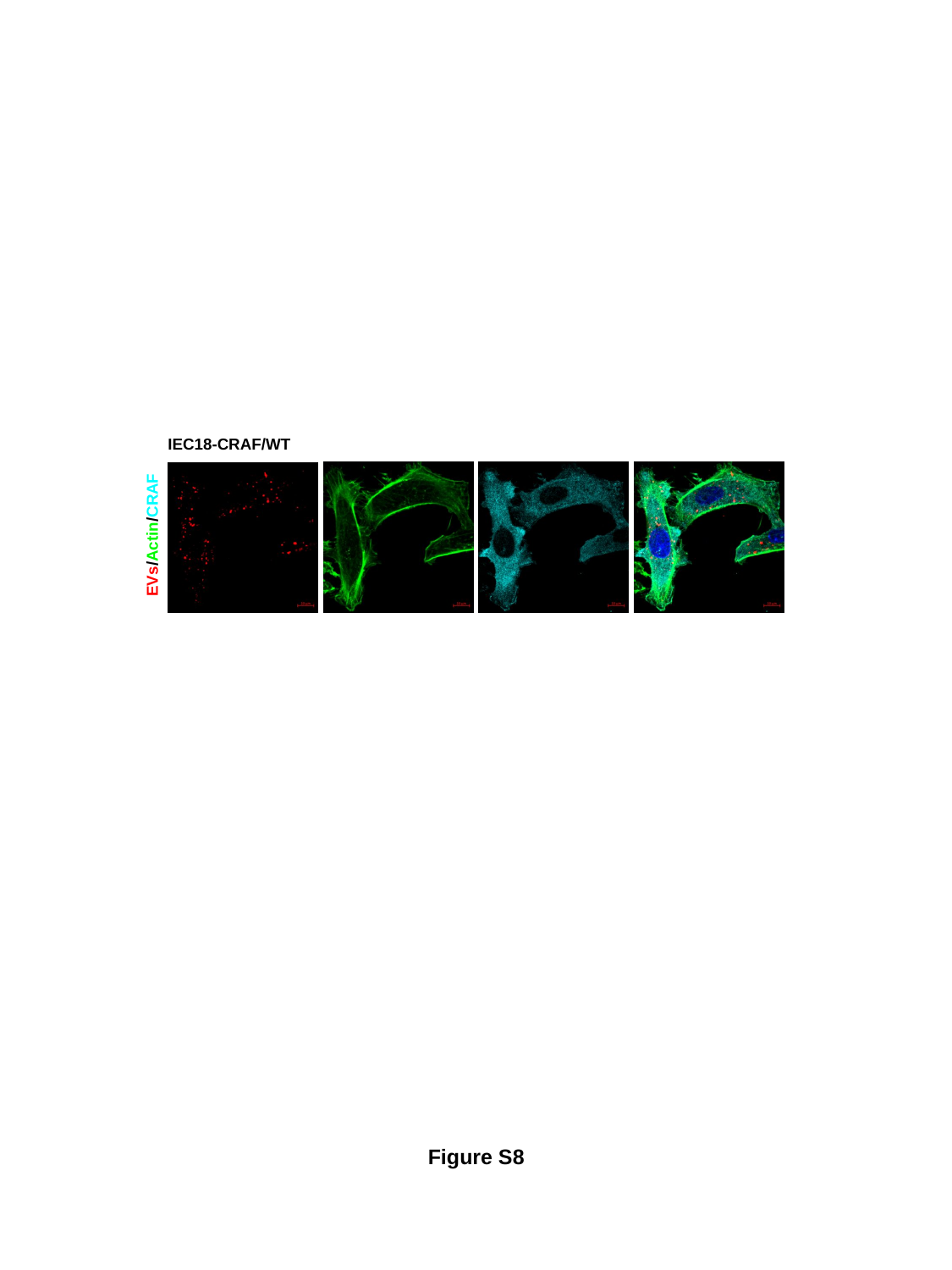

IEC18-CRAF/WT
EVs/Actin/CRAF
Figure S8

## Slide 9
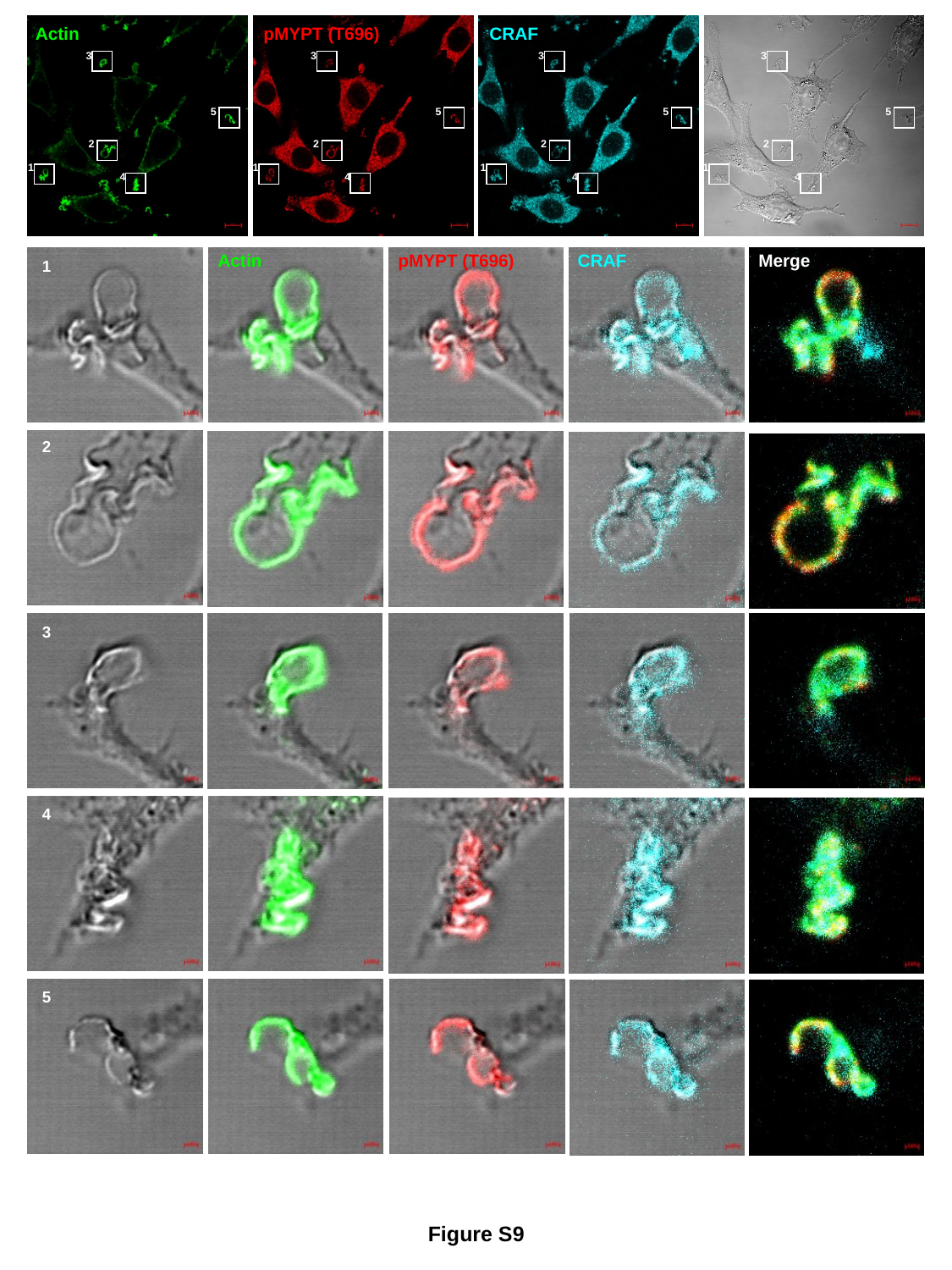

Actin
pMYPT (T696)
CRAF
3
5
2
1
4
3
5
2
1
4
3
5
2
1
4
3
5
2
1
4
Actin
pMYPT (T696)
CRAF
Merge
1
2
3
4
5
Figure S9

## Slide 10
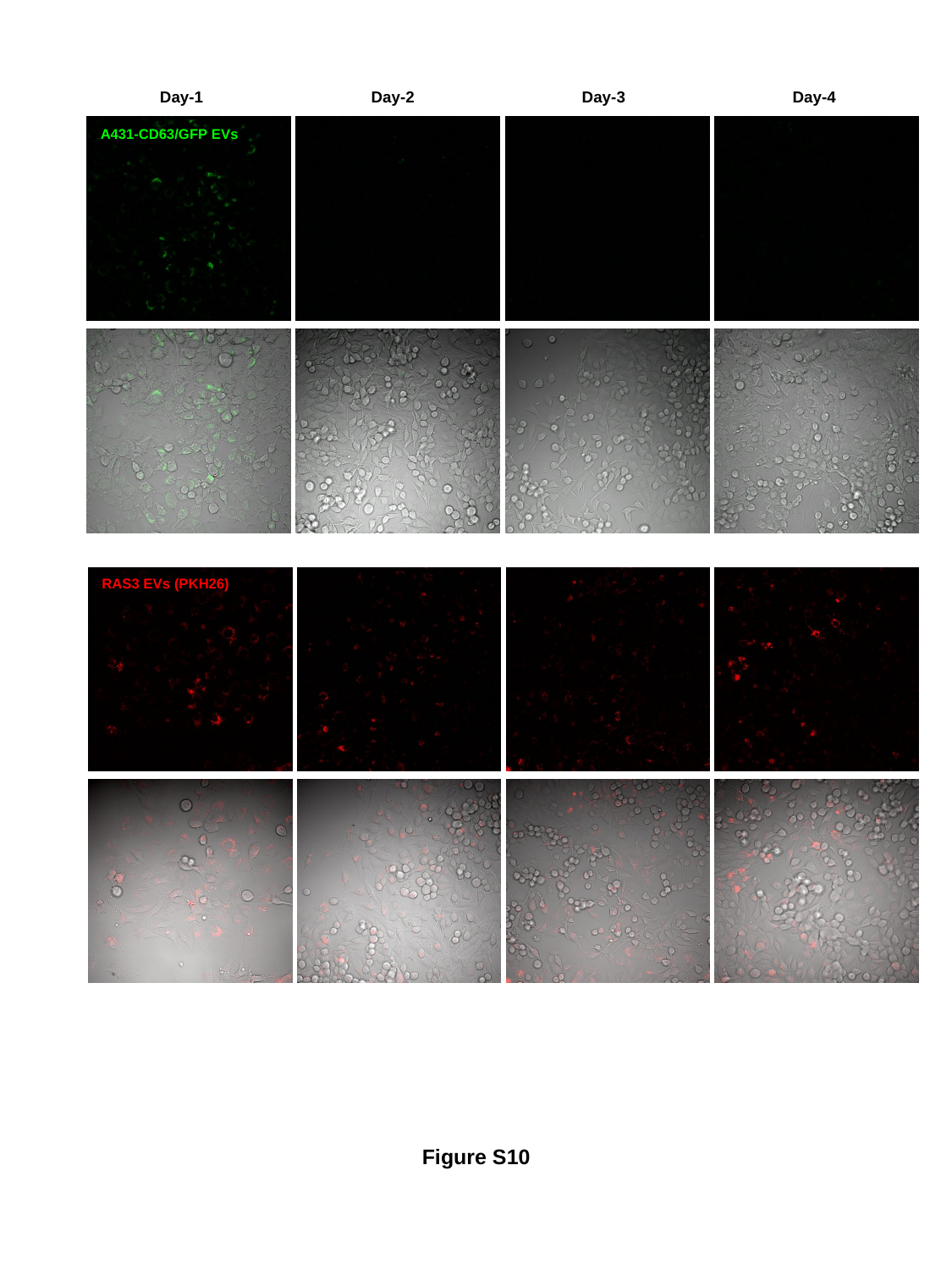

Day-1
Day-2
Day-3
Day-4
A431-CD63/GFP EVs
RAS3 EVs (PKH26)
Figure S10

## Slide 11
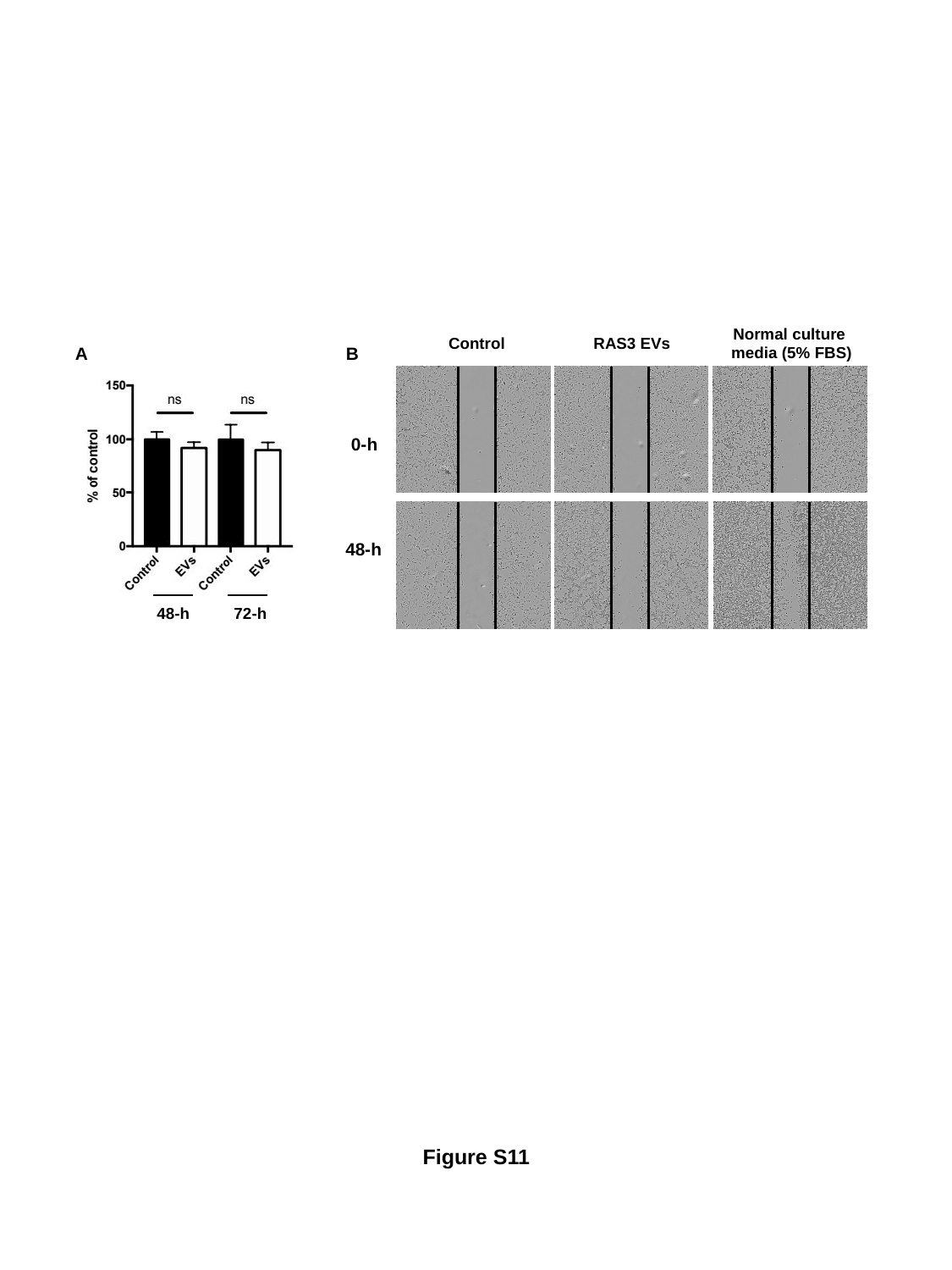

Normal culture
media (5% FBS)
Control
RAS3 EVs
0-h
48-h
A
B
48-h
72-h
Figure S11

## Slide 12
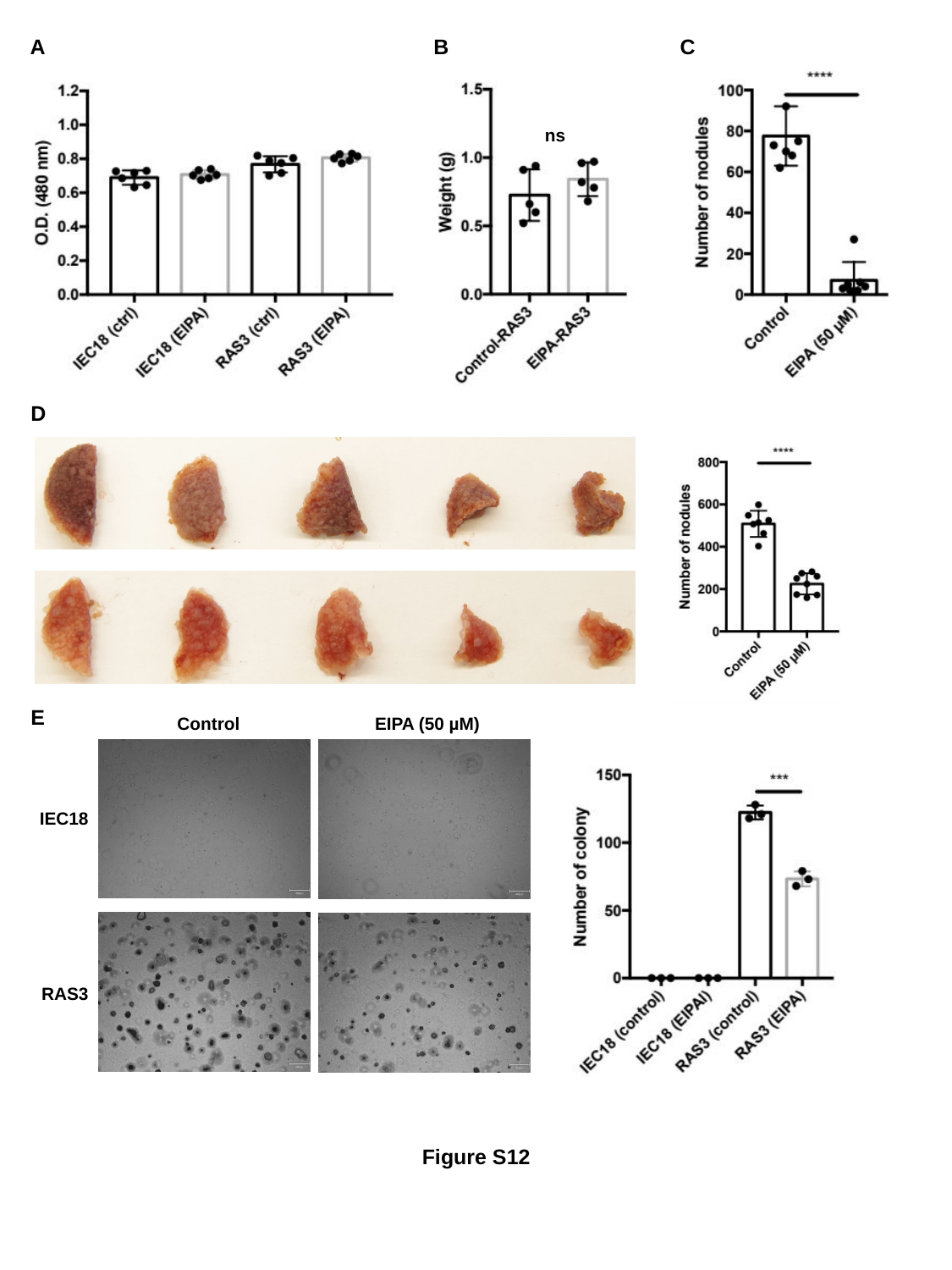

A
B
C
ns
D
E
EIPA (50 µM)
Control
IEC18
RAS3
Figure S12
